# Assessment of Pancreatic β-Cell Function: Review of Methods and Clinical Applications

**DOI:** 10.2174/1573399810666140214093600

**Published:** 2014-01

**Authors:** Eugenio Cersosimo, Carolina Solis-Herrera, Michael E. Trautmann, Jaret Malloy, Curtis L. Triplitt

**Affiliations:** 1 Texas Diabetes Institute, University of Texas Health Science Center at San Antonio, San Antonio, TX 78207, USA; 2 Fueerbarg 16a, D-22393, Hamburg, Germany; 3 Amylin Pharmaceuticals, LLC, San Diego, CA 92121, USA

**Keywords:** β-cell function, DPP-4 inhibitor, euglycemic-hyperinsulinemic clamp, GLP-1 receptor agonist, glucose tolerance test, hyperglycemic clamp, meal tolerance test.

## Abstract

Type 2 diabetes mellitus (T2DM) is characterized by a progressive failure of pancreatic β-cell function (BCF) with insulin resistance. Once insulin over-secretion can no longer compensate for the degree of insulin resistance, hyperglycemia becomes clinically significant and deterioration of residual β-cell reserve accelerates. This pathophysiology has important therapeutic implications. Ideally, therapy should address the underlying pathology and should be started early along the spectrum of decreasing glucose tolerance in order to prevent or slow β-cell failure and reverse insulin resistance. The development of an optimal treatment strategy for each patient requires accurate diagnostic tools for evaluating the underlying state of glucose tolerance. This review focuses on the most widely used methods for measuring BCF within the context of insulin resistance and includes examples of their use in prediabetes and T2DM, with an emphasis on the most recent therapeutic options (dipeptidyl peptidase-4 inhibitors and glucagon-like peptide-1 receptor agonists). Methods of BCF measurement include the homeostasis model assessment (HOMA); oral glucose tolerance tests, intravenous glucose tolerance tests (IVGTT), and meal tolerance tests; and the hyperglycemic clamp procedure. To provide a meaningful evaluation of BCF, it is necessary to interpret all observations within the context of insulin resistance. Therefore, this review also discusses methods utilized to quantitate insulin-dependent glucose metabolism, such as the IVGTT and the euglycemic-hyperinsulinemic clamp procedures. In addition, an example is presented of a mathematical modeling approach that can use data from BCF measurements to develop a better understanding of BCF behavior and the overall status of glucose tolerance.

## INTRODUCTION

**Fig. (1):**
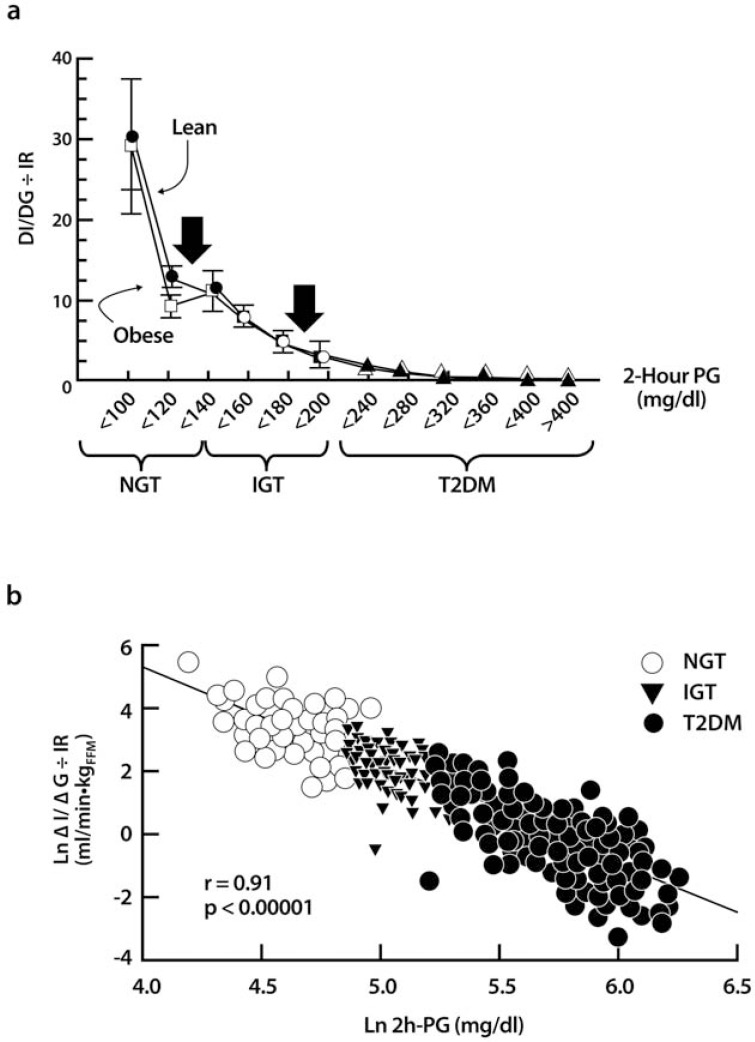
Diagrams illustrating the progressive loss of BCF as glucose tolerance worsens. (**a**) The disposition index (insulin secretion/insulin resistance = ΔI/ΔG ÷ IR) is plotted as a function of the 2-hour plasma glucose concentration (2-h PG) during an OGTT in subjects with a range of glucose intolerance and body weight. If a 2-hour PG <140 mg/dL represents normal glucose tolerance (NGT), subjects in the upper tertile (2-h PG=120-139 mg/dL) have lost two-thirds of their BCF (*left arrow*). Subjects in the upper tertile of IGT (2-h PG=180-199 mg/dL) have lost 80%-85% of their BCF (*right arrow*). Thus, by the time the diagnosis of T2DM has been made, >80% of BCF is gone. Note: Leg-end for y-axis should be "ΔI/ΔG ÷ IR." (**b**) The natural log of the 2-hour plasma glucose concentration (2-h PG) during the OGTT is graphed as a function of the natural log of the disposition index. These two variables are strongly and linearly related (r=0.91; *P*<0.00001). There are no cut points that distinguish NGT from IGT from T2DM. Rather, glucose intolerance is a continuum, and subjects simply move up and down this curve as a function of the disposition index. SI units glucose conversion: mg/dL*0.05551=mmol/L. From reference [2]. BCF, β-cell function; OGTT, oral glucose tolerance test; T2DM, type 2 diabetes mellitus.

β-cell dysfunction with progressive loss of pancreatic β-cell insulin secretion, subsequent to the development of insulin resistance, are key defects associated with the transition from a healthy glycemic state to hyperglycemia, characteristic of untreated type 2 diabetes mellitus (T2DM) (Fig. **1**) [1, 2]. Numerous studies have evaluated β-cell function (BCF) under a variety of metabolic and clinical conditions, and there is strong evidence to indicate that, once the over-secretion of insulin can no longer compensate for the degree of insulin resistance, hyperglycemia becomes clinically significant and the deterioration of the residual β-cell reserve is accelerated. In the natural history of the disease, subjects initially develop postprandial hyperglycemia, followed by fasting hyperglycemia, which can then be clinically detected. The long-term follow-up is usually accompanied by elevated glycosylation of hemoglobin A_1c_ (HbA_1c_) ≥6.5% [1-3]. Prior to overt T2DM (prediabetes), however, individuals with normal glucose tolerance (NGT) develop progressive insulin resistance. As a part of this, there is overproduction of endogenous glucose in the basal state, despite the presence of fasting hyperinsulinemia, and impaired suppression of postprandial glucose (PPG) production, both of which are markers of insulin resistance. Another indicator of insulin resistance is the impaired insulin-mediated glucose uptake and metabolism by the skeletal muscle and other peripheral tissues. As the condition progresses, “at-risk” individuals normally tend to present first with either impaired fasting glucose (IFG), defined by the American Diabetes Association (ADA) as fasting plasma glucose (FPG) from 5.6 mmol/L to 6.9 mmol/L, and/or impaired glucose tolerance (IGT), defined as 2-hour glucose values during an oral glucose tolerance test (OGTT) of 7.8 mmol/L to 11.0 mmol/L [1]. Next, individuals transition to a state in which the pancreatic β-cells are incapable of secreting sufficient insulin to match and overcome the degree of tissue insulin resistance. Clinically, patients with overt T2DM have either HbA_1c_ ≥6.5% or an FPG ≥7.0 mmol/L or a 2-hour post-OGTT or random plasma glucose value ≥11.1 mmol/L. There is always some risk of developing T2DM when these values are abnormal, as described above, and this risk becomes disproportionately greater as the results in subjects approach the higher end of the “normal” spectrum. Some patients may present with classic symptoms of hyperglycemia (or with a hyperglycemic crisis) that, when combined with random sampled plasma glucose ≥11.1 mmol/L, confirm the diagnosis of T2DM. 

Better understanding of the pathophysiology of T2DM has important therapeutic implications [1, 2]. Over time, effective treatment will require a combination of lifestyle interventions and multiple drugs to offset insulin resistance and the progressive worsening of BCF. Ideally, the treatment of T2DM should address these underlying pathologies and not focus solely on HbA_1c_ reduction. Furthermore, evidence suggests that therapy should be started early along the spectrum of decreasing glucose tolerance to prevent progression of the disease with its complications, avoid complete β-cell failure, and reverse insulin resistance. Proof that early interventions are, in fact, the best approach to slow the development of the disease and its progression, and to possibly retard the requirements for exogenous insulin therapy, comprises an active and current topic of clinical investigations. Nevertheless, establishing the value of early therapy necessitates, above all, accurate diagnostic tools for evaluating the underlying status of glucose tolerance in each individual. This review focuses on the most widely used methods for the assessment of BCF within the context of insulin resistance. Several examples of their use and their applications in evaluating the benefits of early diagnosis and the effectiveness of various treatments for prediabetes and T2DM are presented, with an emphasis on the most recent therapeutic options.

## METHODS FOR MEASURING BCF

### Glucose and Insulin Homeostasis During Fasting 

In large population studies, two methods have been utilized for assessing the relationship between glucose and insulin balance under fasting conditions: i) the proinsulin-to-insulin plasma concentration ratio and ii) the homeostasis model assessment (HOMA) [4]. The ratio of proinsulin-to-insulin serves as a surrogate marker of inappropriate intracellular processing of the pro-hormone to insulin and is thus a generic index of BCF (or dysfunction). It is a simple measure to implement, and the calculation is derived from data obtained in routine clinical plasma or serum fasting samples. However, the physiological information this test provides is limited to the steady-state (fasting) condition, and there are insufficient correlations with other BCF tests under a variety of clinical conditions to fully endorse its use in routine practice.

**Fig. (2):**
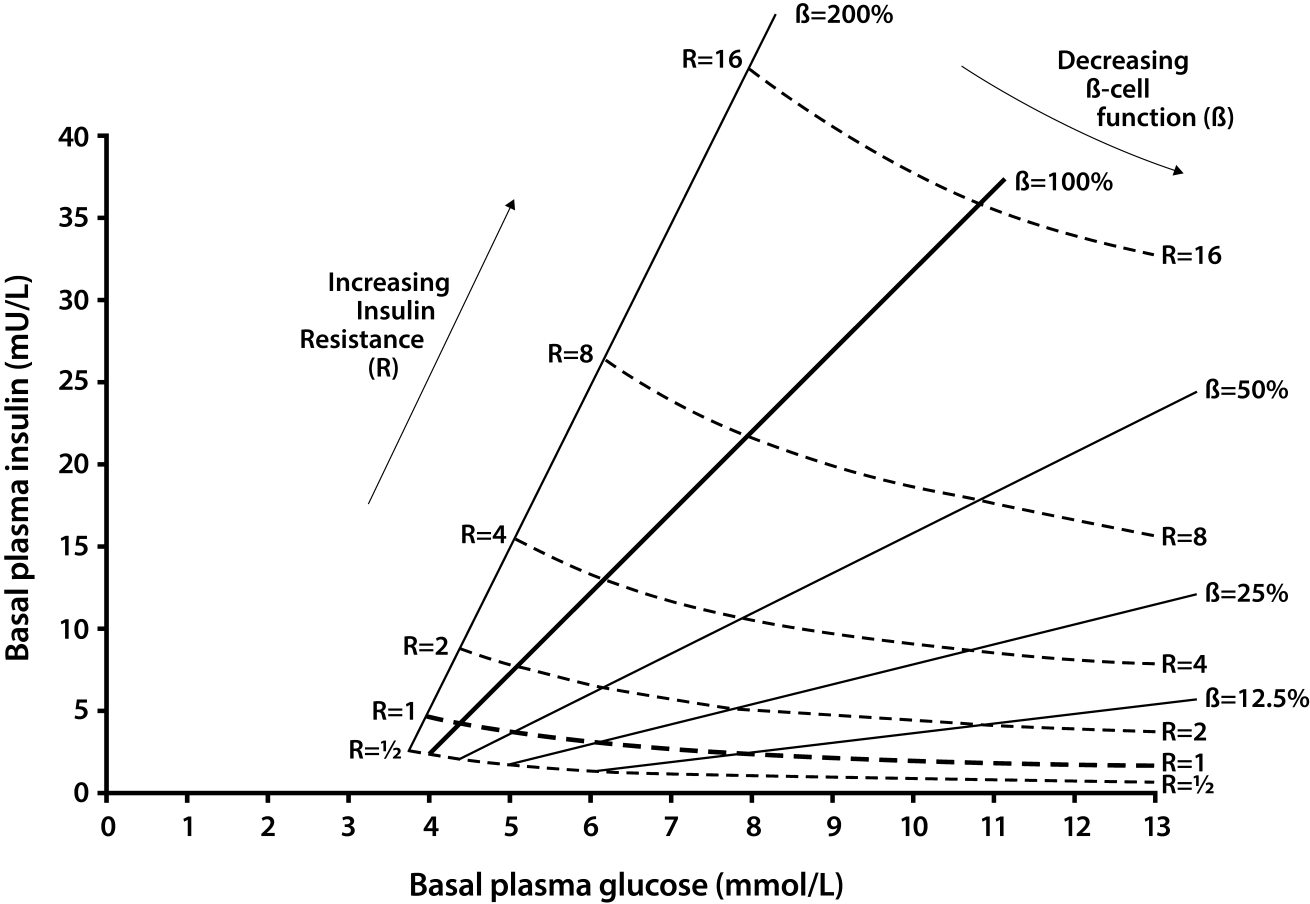
Homeostasis model assessment predictions for the basal or fasting state in humans. The grid shows the model prediction of the steady-state plasma glucose and insulin concentrations for a series of different β-cell functions (solid lines) and insulin resistance values (dotted line). For any individual, fasting observations of plasma glucose and insulin may be entered on the grid and the estimated β-cell function and insulin resistance obtained. From reference [4].

HOMA was first introduced during the seminal United Kingdom Prospective Diabetes Study (UKPDS) where it was used to track the long-term effectiveness of various treatments frequently used in the 1980s, with follow-up of a large population of patients with T2DM [4-6]. HOMA is calculated using steady-state blood concentrations of fasting glucose and insulin to estimate the degree of β-cell deficiency and the target-tissue sensitivity to insulin. After patient data are input, a mathematical model was designed to compute the “idealized” steady-state glucose and insulin concentrations in order to estimate their relative status (Fig. **2**). The feedback loop between the endogenous sources of glucose and the pancreatic β-cell is central to the model: basal plasma glucose concentration is regulated by insulin-dependent endogenous glucose output and plasma insulin concentration is dependent upon the β-cell responsiveness (insulin secretion) to plasma glucose concentration. Thus, HOMA-B assesses BCF by calculating the ratio of fasting insulin-to-fasting glucose concentrations (with empirically determined metabolic conversion factors included in the equation: see Table **1**). A similar calculation using the reverse equation is performed to determine the HOMA-IR, an index of fasting insulin resistance (opposite to the HOMA-S, which stands for insulin sensitivity). By convention, a normal-weight, healthy person younger than 35 years old would have a HOMA-B (surrogate marker of BCF) of 100% and a HOMA-IR (insulin resistance) of 1.0. These calibrations reflect the balance between endogenous glucose production and β-cell insulin secretion, but only during the basal state. HOMA-B correlates well with other methods of assessment of BCF over a wide range of glucose tolerance status (r=0.69 vs. hyperglycemic clamp; r=0.62-0.90 for NGT; r=0.73-0.88 for IGT; r=0.69-0.90 for diabetes) [4, 6]. Similarly, HOMA-IR (HOMA-S) also correlates well with other methods that measure insulin sensitivity (r=0.88 vs. euglycemic-hyperinsulinemic clamp; r=0.58-0.88 for NGT; r=0.83-0.88 for T2DM).

The advantage of HOMA values is that the calculations are relatively simple and derive from parameters typically analyzed during routine clinical and laboratory examinations. The primary strengths of the HOMA approach, therefore, are its “ease-of-use” and relatively low cost, making it particularly suitable for large epidemiology and assessment of clinical treatment studies. In addition, HOMA has been validated against more robust and complex procedures such as the intravenous glucose tolerance test (IVGTT), the hyperglycemic clamp, and the euglycemic-hyperinsulinemic clamp (see below). It has also been updated over time (*e.g.,* HOMA2 [7]) and is available online [8]. However, HOMA-B is an indirect measure of BCF and only takes into account fasting/basal plasma glucose and insulin concentrations. HOMA yields limited information about the daily fluctuations in glucose homeostasis, and the model cannot accurately predict the impact of several common anti-diabetes treatments (*e.g.,* insulin and sulfonylureas [SFUs]) on either BCF or tissue insulin sensitivity. A relatively low precision has been reported for estimates based on the HOMA model (~32% for HOMA-B; ~31% for HOMA-IR) [4]. Perhaps, more importantly, when plasma glucose levels are ≤3.5 mmol/L HOMA estimates cannot be used to assess BCF, because they yield undefined or negative values. Furthermore, the interpretation of results generated when fasting insulin is ≤5 μU/mL (low values typical of most patients with late-stage T2DM) and fasting glucose is <4.5 mmol/L is not valid. Caution is recommended when comparing HOMA values across cultures/ethnicities, because the prevailing "normal" will vary based on differing genetics and environmental factors.

### Dynamic Relationship After Nutrient Load

#### IVGTT

Pancreatic β-cells secrete insulin into the portal vein perfusing the liver, where insulin is partially cleared, prior to entering the peripheral circulation [9-13]. Hepatic insulin clearance rates change following the stimulation of endogenous insulin secretion under both physiological and pharmacological conditions. As a result, the insulin concentration measured in peripheral blood varies and differs from the total amount of insulin secreted by the pancreas under different conditions. The ultimate insulin concentration in peripheral blood represents a balance between the insulin secretory rate and the hepatic clearance rate. Thus, peripheral plasma insulin levels can be reliably used only to compare insulin secretory rates between individuals or groups with known and comparable hepatic clearance rates. In contrast, C-peptide, which is co-secreted with insulin into the portal vein in equimolar amounts, avoids hepatic degradation, and is entirely cleared at a relatively constant rate in peripheral tissues. This differential kinetics has enabled the use of peripheral plasma C-peptide concentrations to more accurately estimate true insulin secretory rates. Subsequently, a sophisticated mathematical model termed "deconvolution of plasma C-peptide concentration" was developed. It takes into account the "constant" rate of peripheral C-peptide clearance to back-calculate the absolute equimolar amounts of insulin secreted endogenously by the pancreas into the portal system. This C-peptide deconvolution analysis is a widely accepted method for accurate and precise estimations of pre-hepatic (total) insulin secretion. There are a few caveats, however. First, C-peptide measurements do not directly quantitate the circulating levels of biologically active insulin. Second, because C-peptide has a relatively long half-life compared to insulin, it will tend to underestimate secretory rates in conditions where insulin release is rapidly changing (*e.g.,* during a quick rise after an intravenous glucose bolus), and it will tend to overestimate secretory rates under conditions where insulin release is rapidly declining.

The standard C-peptide deconvolution model assumes that C-peptide is secreted in a central compartment from which it distributes into a peripheral extravascular compartment [12]. The volume of distribution and kinetic parameters of C-peptide distribution and metabolism were shown to vary by less than 30% in a population highly heterogeneous in terms of age, gender, and degrees of obesity and glucose intolerance. The standardized equation yielded insulin secretion rates differing by only 10%-12% from parameters measured directly on each test subject. Both estimates were tightly correlated, with no systematic under- or overestimation. For the 24-hour mean insulin secretion rate, r=0.935 for normal subjects, r=0.942 for obese subjects, and r=0.941 for patients with T2DM, and for the fasting insulin secretion rate, r=0.904, r=0.927, and r=0.959, respectively. When the effects of body weight, gender, and age were taken into account, the parameters of C-peptide kinetics were very similar in individuals with NGT, obesity, and/or T2DM.

Recently, Tura *et al*. [14] proposed an insulin-based deconvolution model, using standardized insulin kinetic parameters to quantitate pre-hepatic insulin secretion rates, and compared their model with the standard C-peptide deconvolution method [12]. Subjects with varying degrees of glucose tolerance received both an insulin-modified IVGTT and a standard 75-g OGTT [14]. The “insulin deconvolution” method slightly overestimated total insulin secretion rates (85±5 vs. 67±3 nmol) when compared with the C-peptide deconvolution original method (*P*=0.002). This discrepancy was largely attributed to possible variations in the insulin assay. Despite the modest bias, the insulin and C-peptide methods consistently predicted differences between subgroups and relationships with other physiological variables. Both insulin and C-peptide methods yielded similar estimates of the degrees of first-phase insulin response and were superior to the simple determination of plasma insulin concentration. Since the insulin-based insulin secretory rate method compared favorably with the C-peptide approach, it is now accepted as a valid measurement of insulin secretion rates under rapidly changing conditions. Even though further validation studies are warranted, it might be used as a substitute when C-peptide measurements are not available.

**Fig. (3):**
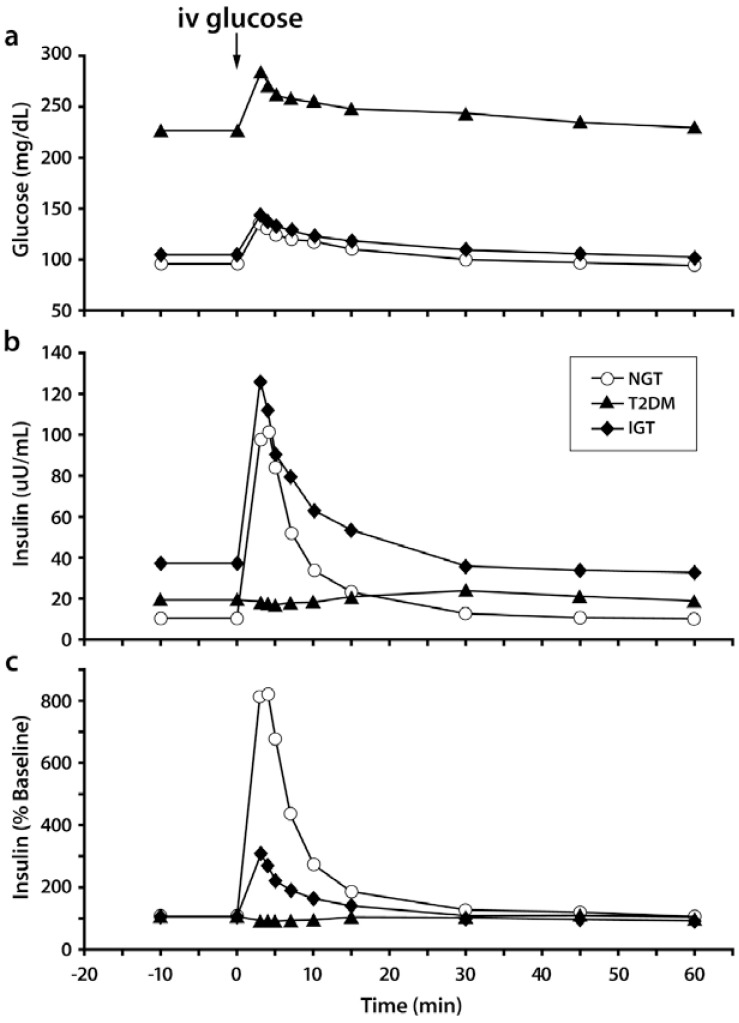
Typical plasma kinetics for glucose (**a**) and insulin (**b**, **c**) during an IV glucose tolerance test in subjects with different degrees of glucose intolerance. Insulin is expressed as a percentage of baseline insulin concentration in panel C. IGT, impaired glucose tolerance; IV, intravenous; NGT, normal glucose tolerance; T2DM, type 2 diabetes mellitus. From reference [184].

The IVGTT with minimal model analysis concomitantly quantifies pancreatic responsiveness and insulin sensitivity [15, 16]. Both first-phase (acute) insulin secretion and second-phase (more prolonged) insulin secretion are estimated. The IVGTT typically requires experienced personnel who are knowledgeable and can perform mathematical modeling. The test typically lasts for 3 hours and is based on principles observed in the 1950s-1960s, namely that adults with NGT respond to the massive hyperglycemia induced by an intravenous or oral glucose bolus with an immediate, maximal secretion of insulin into the circulatory system [17, 18]. The insulinogenic index (IGI) can be derived from data obtained during the IVGTT (RA DeFronzo, personal communication) and OGTT to quantitate this response. IGI is a ratio that relates enhancement of circulating insulin to the magnitude of the corresponding glycemic stimulus. Under test conditions, individuals with NGT have been observed to have a 2-fold or greater insulin secretory response than patients with T2DM (Fig. **3**). Using this approach, it has become evident that the loss of the first-phase insulin response to an intravenous glucose bolus is the earliest defect in BCF detectable in patients with T2DM. The IVGTT and OGTT can generate similar overall magnitude of insulin response, but the dose-response curves reveal kinetically distinct response patterns. This is especially true when plasma glucose concentrations reach different levels following the glucose challenge test.

To perform the IVGTT, subjects are fasted overnight and then infused intravenously with a glucose load given as a bolus to trigger an insulin secretory response [15, 16]. In some cases, an insulin secretagogue other than glucose may be co-administered to maximize β-cell response, depending on study goals. Before, during, and after the glucose infusion, blood samples are collected for measurements of glucose, C-peptide and/or insulin concentration, and other parameters of interest. Models of C-peptide deconvolution or insulin kinetics yield estimates of first- and second-phase insulin responses of the β-cells to the glucose load. Moreover, the use of a glucose kinetics model provides the insulin sensitivity parameter, termed S_I_. This model assumes that glucose acts to increase its own utilization and retard its endogenous production in direct proportion to plasma glucose concentration. Furthermore, insulin is assumed to synergize with these effects of glucose to promote glucose disappearance from plasma dependent upon the insulin concentration. The insulin sensitivity aspects of the minimal model technique have been validated against the standard euglycemic-hyperinsulinemic clamp technique [16, 19].

Tura *et al*. [20] proposed a predictor of the minimal model analysis index using a shorter (1-hour) IVGTT procedure that was validated against the original 3-hour IVGTT and the standard euglycemic-hyperinsulinemic clamp. A calculated S_I_ predictor [CS_I_ = α*K_G_/(ΔAUC_INS_/T)] was based on the calculation of the rate of glucose disappearance (K_G_) and the supra-basal area under the plasma-concentration time curve (AUC) of insulin concentration (ΔAUC_INS_) over an observation period of 40 min (T). The value for α was assumed to be equal to the regression line slope between K_G_/(ΔAUC_INS_/T) and S_I_ in control participants. The CS_I_ and the S_I_ showed high correlation (r^2^=0.68-0.96) and regression line slopes of ~1 in the majority of groups. In patients with T2DM, the CS_I_ tended to overestimate the S_I_. The CS_I _showed good correlation with the *M* value (r^2^=0.82) and an inverse relationship with the body mass index (BMI) associated with the S_I_. The authors concluded that, in situations of low insulin sensitivity (such as T2DM), the CS_I_ may suffer from inaccuracy, but unfortunately the S_I_ may also exhibit inaccuracy under these conditions. The authors also concluded that the shorter test achieved a good approximation of the results obtained with the usual 3-hour IVGTT with minimal model analysis and the insulin sensitivity values derived from a euglycemic-hyperinsulinemic clamp. The obvious advantages of the shorter method are the time requirements and the lower cost when compared to the original, longer IVGTT procedure.

The IVGTT is amenable for use in a routine clinical setting with minimal patient risk and enables, perhaps, the most accurate measurement of the first-phase (acute) insulin secretory response to a nutrient stimulus. However, because most offices dealing with patients who have diabetes are not equipped with the materials and personnel qualified to perform and interpret the results, in our opinion this should not be encouraged. Although it is an artificial, non-physiological parameter, mechanistically, this rapid insulin release (~10 min post-bolus) is almost certainly due to membrane-docked secretory granules within β-cells [19]. The IVGTT avoids the uncertainty and variability of the glucose absorption rates from the gut, which can be highly unpredictable, even in the same subject between repeated procedures. With respect to insulin kinetics and actions, an intravenous procedure provides a simpler foundation for creating models, compared with oral routes of nutrient administration, because the exact amount of injected glucose is known. Keep in mind, however, that the insulin response during the IVGTT is entirely non-physiological. The intravenous route never occurs under normal circumstances and it bypasses the full incretin hormone effect stimulated by oral ingestion, which is now recognized to be of critical importance. Also, the nutrient stimulus is purely carbohydrate in nature. The sensitivity of the β-cell under conditions of hyperglycemia is already altered and any change thereafter will not accurately reflect what could have been to the β-cell response if plasma glucose concentration was near normal. Thus, results obtained when IVGTT is started with fasting hyperglycemia are not interpretable and performing the IVGTT under these conditions is not recommended.

#### OGTT

The OGTT and mixed-meal tolerance test (MTT; *see below*) were designed to assess insulin secretory patterns under more physiological conditions than those of the IVGTT, while capturing the complex interrelationship between glucose levels, insulin secretory response, insulin action, and hepatic insulin extraction [21]. The well-established IVGTT minimal model was used as the basis for the development of a parallel oral minimal model [21-23]. Radioactive glucose tracer protocols have also been used to validate this approach [24-26]. 

**Fig. (4):**
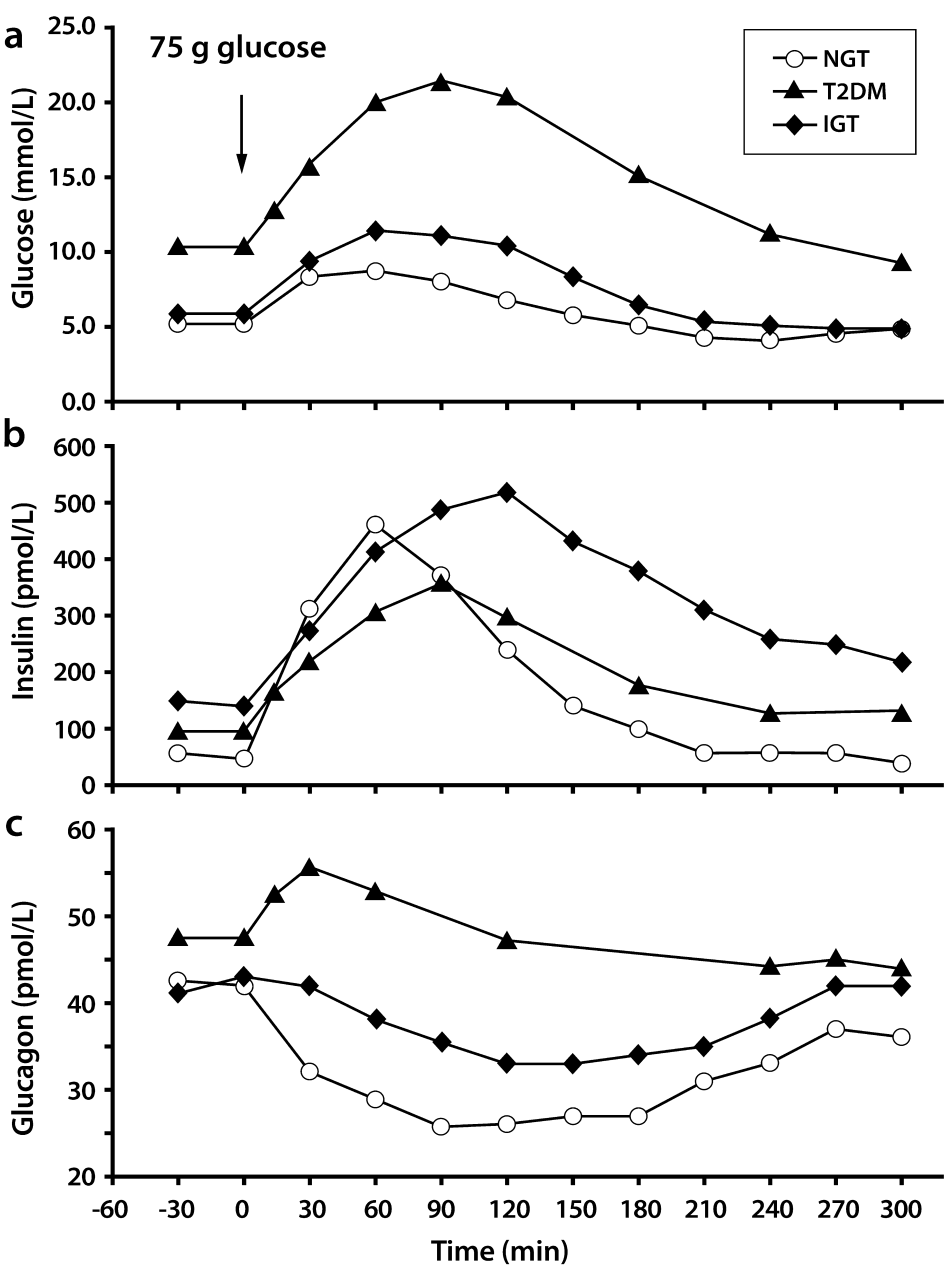
Typical plasma kinetics for glucose (**a**), insulin (**b**), and glucagon (**c**) during a 75-g OGTT in subjects with different degrees of glucose intolerance. Note that the typical OGTT observation period is 120 or 180 min; however, this example covers a 300-min period. IGT, impaired glucose; tolerance; NGT, normal glucose tolerance; OGTT, oral glucose tolerance test; T2DM, type 2 diabetes mellitus. From reference [184].

For the OGTT (Fig. **4**), subjects fast overnight and then quickly ingest an oral glucose load (usually ~75 g) [21]. Before, during, and after the glucose ingestion, blood samples are collected for concentration measurements of glucose, insulin, C-peptide concentration, and other parameters of interest. An estimate of insulin secretion rates may then be calculated from deconvolution of plasma C-peptide concentrations [12]. The primary measures derived from the OGTT are the magnitude and appropriateness of rates of first-phase (acute) and second-phase insulin secretion combined, after the oral glucose load [21]. Typically, these values are captured from the incremental areas under the plasma insulin-time curve (AUC) calculated over the entire 2- or 3-hour test. The first-phase response is typically believed to be embedded in the first 30 min after oral glucose administration, while the second-phase response is more likely to fall between 30-120 (or 30-180) min after the oral glucose load. Because glucose absorption from the gut is an important factor, these patterns cannot be as sharply delineated as the pattern obtained after an IVGTT. Use of a two-compartment oral minimal model can provide two global BCF indexes, namely the insulin secretion rate (ISR) during the OGTT related to increased adiposity and the β-cell index related to glucose tolerance state [23]. In some specific populations, β-cell index may be a more discriminatory indicator than the IGI (Table **1**) for those aspects of BCF related to the control of glucose homeostasis. A more sophisticated variation of the oral minimal model allows for the calculation of the disposition index (DI; Table **1**), in addition to the insulin sensitivity index (ISI) and the ISR [22]. The DI is calculated as the absolute change in plasma insulin divided by the absolute change in plasma glucose multiplied by a measure of insulin sensitivity that can be obtained during the euglycemic-hyperinsulinemic clamp procedure [21].

To accurately measure insulin secretory rates in a given individual under specific circumstances, it is necessary to provide the value in the context of the prevailing relative insulin resistance (*see also euglycemic-hyperinsulinemic clamp below*) [27, 28]. Although the mechanisms underlying the inter-organ relationships between peripheral tissues and the pancreas are not well characterized, the presence of such “cross-talk” is not questioned. Matsuda and DeFronzo [27] validated an ISI derived from the data obtained during an OGTT against directly measured whole-body insulin sensitivity obtained with the euglycemic-hyperinsulinemic clamp technique. In this study, after an overnight fast, subjects underwent a 75-g OGTT and a euglycemic-hyperinsulinemic clamp performed with the infusion of tritiated glucose administered in random order (*i.e.,* OGTT first in some subjects and second in other subjects). The underlying assumptions supporting direct measurement of hepatic insulin sensitivity were that the higher the EGP (primarily hepatic source) and the higher the fasting plasma insulin concentration, the greater the severity of hepatic insulin resistance in the post-absorptive state. Conversely, the inverse of the product of EGP and fasting plasma insulin yields hepatic insulin sensitivity. Furthermore, in the post-absorptive fasting state, most glucose utilization occurs in insulin-independent tissues. Thus, the FPG concentration is largely determined by the rate of basal EGP. By taking into account the observation that suppression of EGP is less complete during an OGTT than during an euglycemic-hyperinsuline-mic clamp in this study, the resulting Matsuda index (MI; Table **1**) from the OGTT was highly correlated with the rate of whole-body glucose disposal during the clamp. The reader is referred to the review by Matsuda [28] for a more detailed discussion of methods for measuring insulin resistance.

The OGTT is simple to administer in routine clinical practice. The full incretin hormone effect is stimulated by the oral ingestion, giving the OGTT a greater physiological significance than the IVGTT. However, it shares the same weakness in that the nutrient stimulus is purely carbohydrate in nature.

#### MTT

Of the three dynamic tests routinely used to assess glycemic control after a nutrient challenge (OGTT, MTT, and IVGTT), the MTT most closely tracks the physiological responses expected to occur during an individual’s normal day-to-day life. During the MTT, as with the OGTT, the full incretin hormone effect is tested following the oral ingestion of nutrients [29, 30]. Although the MTT is difficult to standardize and is more cumbersome to administer than the OGTT, it is of greater physiological significance. During the MTT, the body responses to a normal load of mixed nutrients absorbed from the gut at differing rates are tested. After an overnight fast, subjects ingest a pre-established calorie load in the form of a mixed solid/liquid meal within a specified time period [24-26, 31]. Prior to, during, and for 2-8 hours after nutrient ingestion, blood samples are collected for measurements of glucose, C-peptide, glucagon, and/or insulin concentration, as well as other parameters of interest. The determination of plasma C-peptide concentrations during a 180- to 360-min MTT can be used to calculate ISR, using the deconvolution analyses. This ISR can be further divided by an index of insulin resistance acquired for the same individual during a euglycemic-hyperinsulinemic clamp. This yields a value for insulin secretion *in vivo* that is similar to the “gold standard” for the determination of insulin secretion capacity known as the DI. As an alternative, insulin sensitivity values can also be derived from the MTT [31, 32], an approach that has been validated with radioactive tracers against the IVGTT, against a reference meal model, and against the euglycemic-hyperinsulinemic clamp [22-24].

The two-compartment model of C-peptide kinetics during an MTT, after deconvolution, was first proposed by Hovorka *et al.* [31]. This approach quantitates pancreatic insulin secretion following the meal by assuming a linear relationship between C-peptide secretion rate (normalized to the C-peptide volume of distribution) and the plasma glucose concentration. The model derives a BCF index (M_I_) that is the increment in C-peptide secretion rate caused by a unitary increase in plasma glucose over basal values. In other words, M_I_ captures the ability of PPG excursions to stimulate the pancreatic β-cell. The model also yields an estimate of basal (M_0_) β-cell sensitivity. In other words, M_0_ captures the ability of FPG concentrations to stimulate the pancreatic β-cell. Both M_I_ and M_0_ are normalized to the C-peptide volume of distribution within the central (plasma) compartment, resulting in indices that represent ISRs per unit volume of plasma. Thus, M_I_ is a composite index, and its primary strength is in providing an overall measure of β-cell responsiveness after a meal. However, M_I_ does not allow for separate analysis of the net glucose effect and the incretin effect. Meal size and composition may also affect M_I_ and M_0 _values, thus limiting this approach to being used for comparisons between small groups and study populations under similar experimental conditions.

Dalla Mann *et al*. developed an MTT-based model that estimated the rate of glucose appearance and insulin sensitivity [24-26, 32]. This model coupled a single-compartment minimal model with a parametric description of glucose appearance rate. This approach was developed to estimate the rate of glucose appearance in plasma after a meal and the sensitivity of the β-cell response to that glucose (S_I_) under physiological conditions. Thus, S_I_ is a composite index of insulin action on both glucose production and overall disposal. A radioactive tracer was added to the meal to specifically measure the ability of insulin to stimulate glucose disposal. This allowed for discrimination between glucose production, including gut absorption rate over time, and overall glucose disposal. When data from tracer and non-tracer oral minimal modeling were combined, ISRs, insulin sensitivity, and BCF indices could all be derived from the MTT.

### Metabolic Clamp Procedures 

#### Hyperglycemic Clamp With/Without Arginine Stimulation

**Fig. (5):**
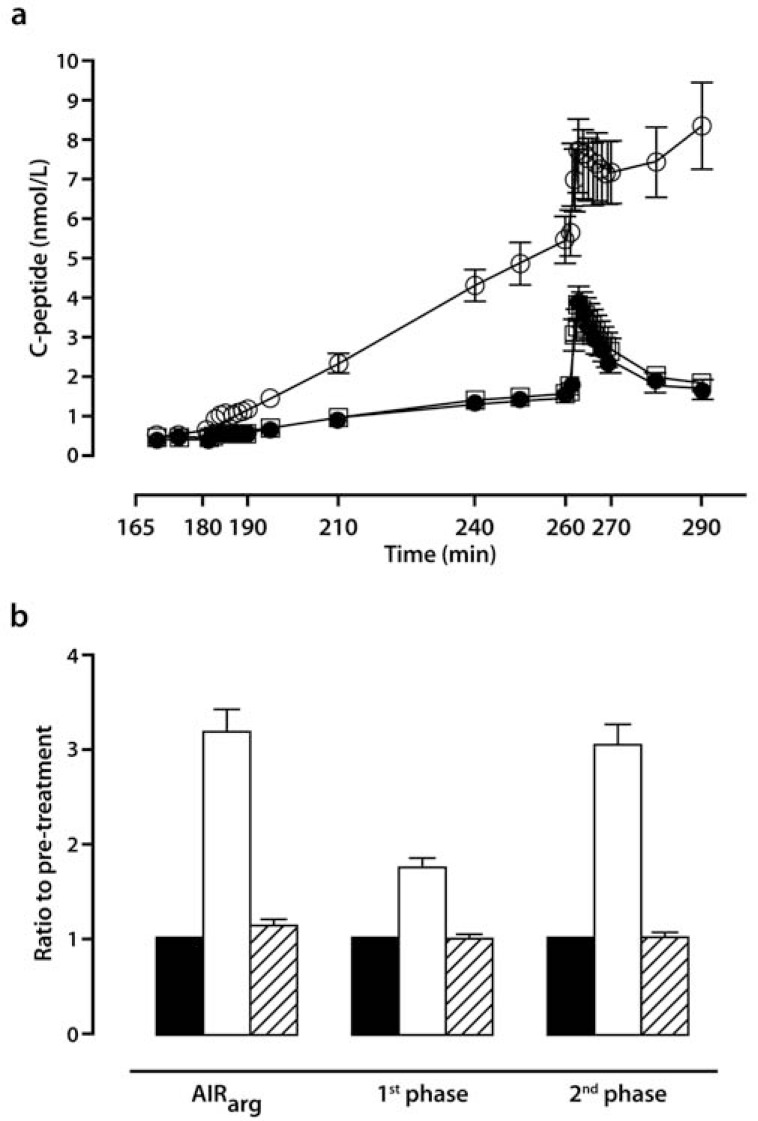
Representative illustration of a hyperglycemic clamp with 15 mmol/L arginine stimulation at the 260-min time point. (**a**) Plasma C-peptide concentrations. Filled circles, baseline. Open squares, Week 52. Hatched squares, Week 56. Mean±SEM (**b**) Ratios to pretreatment for AIR_arg_, first-phase secretory response, and second-phase secretory response in patients with type 2 diabetes treated with exenatide 10 µg twice daily for 52 weeks, followed by no drug treatment for 4 weeks. Filled columns, baseline. Open columns, Week 52. Hatched columns, Week 56. Geometric mean±SEM. AIR_arg_, acute plasma insulin response to arginine; SEM, standard error of the mean. From reference [132].

The hyperglycemic clamp provides a very reproducible technique for measuring BCF under maximal stimulatory conditions [33] (Fig. **5**). Typically, the plasma glucose concentration is acutely raised to 6.9 mmol/L above basal or to a set glucose value by a priming intravenous infusion of glucose, followed by a continuous intravenous glucose infusion. The desired hyperglycemic plateau is subsequently maintained by adjustment of a variable glucose infusion, based on frequent plasma glucose measurements and the negative feedback principle. Because the plasma glucose concentration is held constant, the glucose infusion rate becomes an index of glucose metabolism. In non-diabetic, normal subjects and under the imposed conditions of constant hyperglycemia, the plasma insulin response is typically biphasic with an early burst of insulin release during the first ~10 min (first phase), followed by a gradually progressive increase in plasma insulin concentrations (second phase) up to a peak, and then a gradual decrease to baseline after the clamp is released. The second phase will continue to gradually rise over time as long as the hyperglycemic stimulus remains. The maintenance of the same steady-state glucose concentration in all subjects obviates the need for indices such as insulin/glucose ratio. In addition to assessment of first-phase insulin release from C-peptide deconvolution, the hyperglycemic clamp is often used to evaluate non-glucose insulin secretagogues, such as arginine. This is tested during the interval at the end of the 180-min clamp, with the caveat that it is also an artificial and non-physiological test, although it provides the maximum response value of insulin secretion for any given individual. Maximal stimulus occurs when plasma glucose levels are held for more than 30 min above 450 mg/dL prior to the arginine bolus. One can also perform a stepped clamp with arginine testing to evaluate the half maximal response (see Polonsky *et al.* [9] for more details). GLP-1 is another example of a non-glucose insulin secretagogue that has been used in this manner [34].

When using the hyperglycemic clamp to compare results amongst different diabetic and non-diabetic populations, it is very important that the fasting glucose concentrations at the start of the test be equal (or near-equal). This is an essential requirement because baseline plasma glucose, regardless of the value, already provides a stimulus in and of itself for basal insulin secretion. All changes further induced by an acute elevation of plasma glucose thereafter should reflect a stimulus to the pancreatic β-cells arising from identical hyperglycemic stimuli, as long as equivalent basal conditions are matched. If not equal, baseline stimuli to pancreas will start at different levels (either in different study days or in different research subjects) and additional changes in insulin secretion will not reflect an accurate match as an index of pancreatic insulin reserve. For example, an individual with good glycemic control, indicated by a fasting glucose level of 5.6 mmol/L, will have a different physiological response to a forced acute glucose elevation of 2.8 mmol/L (final value, 9.7 mmol/L) than would a diabetic individual with a starting fasting glucose level of 11.1 mmol/L (final value, 15.3 mmol/L) in response to an identical plasma glucose elevation of 2.8 mmol/L. It is worth mentioning, however, that some, but not all, diabetic individuals may retain maximal insulin secretory capacity early on in the disease process, which can be detected by functional studies. 

Bonadonna *et al*. [35] adapted a minimal model to assess BCF during a hyperglycemic clamp and validated the model against data from the OGTT. C-peptide kinetics were based on population-derived parameters [12]. Both procedures yielded good data fits when assessed by the weighted-residual methods. Minimal model analysis of data from the standard OGTT and the hyperglycemic clamp revealed distinct relationships between BCF during the OGTT and first- and second-phase insulin response during the hyperglycemic clamp versus plasma glucose concentrations and insulin sensitivity. The data utilized in the development of this model best fit a linear correlation with a negative slope between first-phase response and fasting glucose. By contrast, the data best fit a power function between second-phase response and insulin sensitivity.

The primary strength of the hyperglycemic clamp is its high reproducibility as a method of assessing β-cell sensitivity to glucose. This technique enables follow-up of any active improvement or deterioration in the pancreatic insulin response to changes in plasma glucose levels over time in the same subjects, as well as in longitudinal population studies. This procedure also measures, and is a strong indicator of, the modifications in ISRs during a variety of medications used in patients with diabetes. This clamp procedure is technically demanding, requires advanced skills and trained personnel, and is difficult to perform, not to mention its high cost. The technique assumes that noninsulin-dependent glucose uptake is unaffected by the pathological state, an assumption that is not always true. Moreover, data interpretation is limited by the requirement of a near-equal starting basal plasma glucose level. Comparisons are more reliable if they are made among subjects or across populations, or even in the same subject in two different occasions, when baseline glucose concentrations are equivalent. If starting basal plasma glucose levels are unequal, an intravenous insulin infusion overnight becomes necessary in an effort to eventually reach an isoglycemic pre-determined baseline glucose concentration. More technical difficulties are added, and this may further confound the interpretation of the results obtained and add to the overall cost of the procedure.

#### Euglycemic-Hyperinsulinemic Clamp

The euglycemic-hyperinsulinemic clamp is not designed to directly evaluate BCF, but rather provides insulin sensitivity parameters that can be utilized for a more accurate and precise assessment of other BCF measures [33]. It is often used to derive values pertaining to insulin resistance and for assessing overall metabolic status, both of which can be helpful in estimating the residual BCF. For example, the* M *value derived from this clamp procedure gives an estimate of total body glucose metabolism by assuming that basal hepatic glucose production is suppressed by the infusion of glucose and insulin during the clamp. This assumption is true for a standard insulin infusion dose, especially in healthy non-diabetic subjects, whereas higher insulin doses are needed in patients with T2DM to completely suppress EGP. The *M* value is usually calculated as the average value obtained during the steady-state concentration period (frequently the last 40-60 min of a euglycemic clamp). The time course of the amount of glucose metabolized by the body can be quantified using a combination of the *M* value and the steady-state plasma insulin concentration. The* M/I *ratio derived from the euglycemic-hyperinsulinemic clamp provides an estimate of the amount of glucose metabolized in peripheral tissue and a measure of tissue sensitivity to insulin during steady-state hyperglycemic conditions. Use of the *M/I* ratio assumes that hyperglycemia *per se* does not enhance glucose uptake, which is not always the case.

During a euglycemic-hyperinsulinemic clamp, a variable glucose infusion is used to maintain a "normal" pre-specified value of plasma glucose concentration in combination with a constant rate of insulin infusion that produces hyperinsulinemia [33]. The standard clamp procedure involves acute elevation and maintenance of plasma insulin concentration at ~100 μU/mL. The variable glucose infusion is adjusted at regular intervals using frequently obtained plasma glucose concentrations and a negative feedback loop. When used in combination with isotope tracers, this test can provide an estimate of the ability of insulin to suppress EGP, a measure of hepatic insulin resistance, and also the total amount of insulin-mediated glucose utilization. Without the addition of isotope tracers, under steady-state conditions of euglycemia with hyperinsulinemia, the glucose infusion rate equals glucose uptake by all the tissues in the body, and this represents a measure of tissue sensitivity to insulin. 

The primary strengths of the euglycemic-hyperinsulinemic clamp are that i) it provides a direct measure of whole-body insulin sensitivity and ii) it enables the distinct assessment of hepatic and peripheral insulin sensitivity in the same individual. Also, it helps to quantitate the total amount of glucose being metabolized. This procedure can be performed using a wide range of insulin dose infusion rates, and even in a stepwise sequence to assess different effects on glucose metabolism of increasing levels of plasma insulin, or it can be used as a standalone single insulin dose test. Keep in mind, however, that during the euglycemic-hyperinsulinemic clamp procedure, glucose and insulin concentrations are artificially manipulated and may reach levels not observed under physiological conditions. These are in sharp contrast to the IVGTT, OGTT, and MTT, in which both glucose and insulin plasma concentrations vary widely, but often within the physiological range (OGTT and MTT). Thus, the dynamic relationship between these parameters is frequently preserved. At the conclusion of the euglycemic-hyperinsulinemic clamp, the *M*/*I* ratio is calculated under the assumption that hyperglycemia *per se* does not enhance glucose uptake, which is not true in most circumstances. Use of this clamp technique assumes that noninsulin-dependent glucose uptake is unaffected by the underlying pathological state being studied. In addition, the euglycemic-hyperinsulinemic clamp is cumbersome to perform and requires advanced technical skills. It is not cost- or time-effective to be employed in studying interventions in large groups of subjects.

### Mathematical Modeling of Physiological Data from any BCF Methodology

The Mari/Ferrannini model is presented here as an example of a mathematical modeling approach that can use source data from the IVGTT, OGTT, MTT, and metabolic clamp procedures to develop population-wide conclusions concerning the overall status of BCF and glucose tolerance [36, 37]. This model incorporates several factors that have been shown to play a major role in regulating the pancreatic β-cell’s secretory response to glucose stimulation. The model describes BCF independent of the confounding influence of differing plasma glucose concentrations among subjects with differing disease status (*e.g.,* normoglycemic, glucose intolerant, or diabetic subjects) and/or those receiving differing treatments. 

In this model, the investigators proposed the incorporation of the following factors: 1) the glucose sensitivity as a reflection of the ability of the β-cell to respond to changes in prevailing plasma glucose concentrations; 2) the rate sensitivity that refers to the magnitude of the β-cell response to a given rate of change in plasma glucose concentration; and 3) the potentiation factor that is related to the release of endogenous incretin hormones, neuronal inputs, and changes in the incremental plasma glucose concentrations after ingestion of a meal. All of these factors are directly involved in determining the sensitivity of the β-cell insulin secretory response to any change in plasma glucose concentrations.

The main strength of this approach is that it allows for the exploration of the dynamic relationship between ambient glucose, ambient insulin concentrations, and β-cell response over time. Some weaknesses include the fact that the selection of data entered into the model is somewhat subjective, there is no widely distributed and validated computer program yet, and it is inherently difficult to understand the complex mathematics underlying the model. As a result, this model is nearly impossible to apply in routine clinical settings, unless the assistance of an expert in the field or a validated simple computerized program is available.

## SPECIFIC CLINICAL APPLICATIONS OF BCF ASSESSMENT

Therapies that optimize glycemic control can reduce most of the complications and poor outcomes associated with morbidities and premature mortality in patients with T2DM [1, 38]. Unfortunately, glycemic control is difficult to achieve and maintain because of the progressive nature of the disease, suboptimal patient adherence to treatment regimens, lack of adequate healthcare provider education, limited availability of pharmacological agents that fully meet all the pathophysiological defects of the disease, and the risk for detrimental side effects (*e.g.* hypoglycemia, edema, weight gain) with some commonly used therapeutic agents.

### BCF Across the Spectrum From NGT to Overt T2DM

Treatments that prevent or delay the onset of T2DM in individuals with IFG and/or IGT (prediabetes) could have significant long-term benefits to both patients and the healthcare system. In the United States, some have predicted that approximately 40%-50% of individuals with prediabetes will progress to T2DM [39]. Individuals with prediabetes, including those with mildly elevated HbA_1c_, are at a relatively high risk for the future development of not only T2DM, but also cardiovascular disease (CVD), dyslipidemia, hypertension, and stroke [1, 38]. For example, in a systematic review of studies with a mean follow-up of 5.6 years, individuals with an HbA_1c_ between 5.5% and 6.0% had a substantially increased risk of T2DM (5-year incidence in the general population from 9% to 25%). As HbA_1c_ increased, so did the 5-year risk of developing T2DM (*e.g.,* 25%-50% for HbA_1c_ values of 6.0%-6.5%) [40]. Recently, Kanat *et al*. [41] reported that, as HbA_1c_ rose above 6.0%, both BCF and insulin sensitivity decreased markedly. In this study, participants were assessed using an OGTT followed by C-peptide deconvolution for ISR, calculation of the MI for insulin sensitivity, and comparison of relative BCF to insulin resistance using the DI. At HbA_1c_ values below 5.5%, both the MI and the DI remained constant. However, as the HbA_1c_ increased above 5.5%, both of these indices decreased, suggesting worsening insulin-mediated glucose metabolism simultaneous with progression to β-cell failure. Using these methods, subjects with NGT and HbA_1c_ values <5.7% were shown to have BCF comparable to that of subjects with NGT and an HbA_1c_ value ranging from 5.7% to 6.4%. However, subjects with IFG (ADA 2003 definition of FPG = 6.1-6.9 mmol/L) or IGT had decreased BCF regardless of HbA_1c_ level. Overall, at an HbA_1c_ of 6% there was a 62% decrease in the DI compared with an HbA_1c_ <5.7%. Current ADA guidelines recommend that patients with IGT, IFG, or HbA_1c_ of 5.7% to 6.4% should be treated with lifestyle interventions with the goal of losing at least 7% of body weight [1]. The use of low-dose metformin (MET) can also be considered for this group at high risk for the development of T2DM as part of preventative measures. This is particularly useful in patients with a BMI >35 kg/m^2^, those aged <60 years, or women with prior gestational diabetes. Additional examples of epidemiological studies are presented in (Tables **2** and **3**). 

According to recent studies, individuals in the upper tertile of IGT are maximally/near-maximally insulin resistant, have lost 70%-80% of their pancreatic β-cell response, and already have approximately a 10% incidence of diabetic retinopathy [2, 41]. Pharmacological interventions that reverse β-cell dysfunction and/or insulin resistance have been shown to prevent or delay the progression from prediabetes to overt T2DM. For example, MET reduced the development of T2DM by 31% in the US Diabetes Prevention Program study, presumably through its indirect effects to reduce β-cell workload (inhibition of hepatic glucose production and improved hepatic insulin sensitivity). Treatment with a thiazolidinedione (TZD) has also been shown to reduce conversion from IGT to frank diabetes [42, 43]. In the Diabetes Reduction Assessment with Ramipril and Rosiglitazone Medication (DREAM) study, rosiglitazone (ROSI) reduced the rate of conversion from IGT to T2DM by 62%, and in the Actos Now for the Prevention of Diabetes (ACT NOW) study, pioglitazone (PIO) reduced the rate of IGT conversion to T2DM by 72%. In both cases, TZD-enhanced BCF was a strong predictor of drug effectiveness. Because of the edema, body fat redistribution, body weight gain with excess fat accumulation, and high cost associated with currently available TZDs, this agent class has been relegated to a last resort in the prevention of T2DM. Early data also support a role for glucagon-like peptide-1 receptor agonists (GLP-1RAs) in delaying the conversion from prediabetes to T2DM [2]. For example, 1 year of treatment with the GLP-1RA liraglutide (LIRA) decreased the proportion of subjects with prediabetes as compared with placebo injection or oral orlistat, an inhibitor of pancreatic lipolytic enzymes in the gastrointestinal tract that reduces meal fat absorption [44]. LIRA also decreased the proportion of subjects with prediabetes significantly compared with orlistat at the end of year 2 (*P*<0.001). At the end of year 2, 52%-62% of prediabetic subjects treated with varying doses of LIRA achieved NGT versus 26% of those treated with orlistat. Unfortunately, no measures of BCF were reported for this study, although improved BCF might be inferred based on the substantial weight loss that occurred and the known mechanisms of action of GLP-1RAs and orlistat (*see GLP-1RA section below*).

### Effects of Various Treatments on BCF in Patients with T2DM

#### Lifestyle Interventions

In individuals without overt T2DM, obesity is an insulin-resistant state associated with hyperinsulinemia and a modest expansion of β-cell mass, estimated to correspond to an increase of 10%-30% for each 10 kg of excess body weight [45]. Peripheral hyperinsulinemia is the combined result of insulin hypersecretion and reduced insulin clearance. For equivalent degrees of insulin resistance, non-diabetic subjects with different BMIs have different insulin delivery rates to the systemic circulation in the fasting state. This modeling program predicted an increase in the insulin delivery rate of ~4 pmol/min for each BMI unit increase or for each 10-μmol*min^-1^/(kg of free fat mass) decrease in insulin sensitivity. This dual dependence was not modified by FPG concentration. Furthermore, increases in fasting ISR of 12 pmol/min for each unit increase in BMI and of 20 pmol/min for each 10-μmol*min^-1^/(kg of free fat mass) decrease in insulin sensitivity were also predicted. After nutrient ingestion (OGTT, MTT) or during an IVGTT, total insulin output was predicted to increase by 16 nmol for each 10-μmol*min^-1^/(kg of free fat mass) decrease in insulin sensitivity. Conversely, weight loss is usually associated with improved whole-body insulin sensitivity. Morbidly obese individuals have been found to respond to post-gastroplasty weight loss with an increase in DI. In obese patients with T2DM, weight loss is usually followed by a decrease in insulin resistance and an increase, rather than a decrease, in β-cell responsiveness with reported cases of reactive hypoglycemia that might be due to lowered glucotoxicity and lipotoxicity [45, 46]. As a consequence, weight loss is, in most cases, accompanied by improved diabetes control.

Obesity is one of the fundamental factors underlying the current epidemic of T2DM [38, 47, 48]. For patients with T2DM, weight loss of as little as 5%-10% can improve glycemic control, dyslipidemia, and hypertension, and reduce the need for anti-diabetes drugs [49]. Body weight loss in overweight/obese patients with T2DM has been associated with a 25% reduction in total mortality and a 28% reduction in mortality from CVD and diabetes [50]. Numerous clinical investigations have demonstrated the ability of lifestyle interventions, such as dietary changes and increased physical activity, to improve BCF, insulin resistance, and glycemic control, at least in the short term (Tables **2** and **3**) [1, 9, 49-56]. Unfortunately, almost all of the lost weight is regained within 5 years in most cases [49], and additional strategies, including more intense physical activity, revised dietary manipulations, and medications known to protect the pancreas and/or improve tissue insulin sensitivity, become necessary to maintain glycemic control [1]. 

Lifestyle interventions are considered first-line therapy for prediabetes and newly diagnosed patients with T2DM [1]. Data from large studies indicate that restoration of glycemic control, when coupled with weight loss, can improve hypertension, dyslipidemia, and other CVD risk markers [1, 57-60]. In the Look AHEAD (Action for Health in Diabetes) trial, 1 year of intensive lifestyle intervention in patients with T2DM yielded significant reductions in mean body weight of 8.6% and mean HbA_1c_ of -0.7%, accompanied by improvements in blood pressure, triglycerides, and high-density lipoprotein cholesterol (HDL-C) [57]. In the 10-year Belfast Diet Study, the patient population was enrolled in a program of intensive diet therapy until such time as they crossed predetermined thresholds for loss of glycemic control and weight gain, resulting in a switch to other therapies [53]. Out of the 420 enrolled patients, 173 remained on diet therapy for the entire 10 years. Analyses were conducted on the groups of patients who were divided into four cohorts on the basis of the date of failure of diet therapy. Following the initial reductions in body weight during the first months of treatment, weight remained relatively constant in all patients, independent of the time-to-diet failure. The rate of rise in FPG levels was inversely related to duration of successful diet therapy. HOMA-B was assessed for the first 6 years of the study and over this time course the pattern of HOMA-B fall mirrored the progressive rise in FPG concentration in each of the four cohorts, with the least HOMA-B worsening in the cohort that was able to maintain diet therapy the longest (*P*<0.05). This observation is not surprising because the results are a direct consequence of the formula used to calculate HOMA-B and there was no change in insulin sensitivity (HOMA-S) across cohorts regardless of the “time to diet failure.” Moreover, the findings are most likely due to the immediate removal from the study cohort of any patient losing glycemic control. Successful continuation on diet therapy was significantly associated with a trend across tertiles for lower FPG (*P*<0.0001), higher BCF as assessed by the OGTT performed 6 months after enrollment (30-min ΔI/ΔG; *P*<0.0001), and increasing age (*P*<0.01). In summary, patients who were able to continue on diet alone for longer periods during the 10-year observation interval had lower ongoing FPG levels and better BCF. Further examples of lifestyle intervention studies can be found in (Tables **2** and **3**).

#### MET in Patients with T2DM

MET is an oral antidiabetic drug (OAD) that reduces hyperglycemia in patients with T2DM by inhibiting hepatic glucose production (*i.e.,* by improving hepatic insulin sensitivity) and by slowing glucose absorption from the gut [2, 61]. MET may also have a mild effect to enhance insulin sensitivity and glucose utilization in peripheral tissues. Together, these mechanisms result primarily in decreased FPG with subsequent reductions in PPG concentrations. Because MET presumably works through a cellular cAMP-kinase signal transduction pathway in the liver, this drug does not augment pancreatic insulin secretion. As a result, MET treatment has a low risk of inducing hypoglycemia in patients with T2DM. Overall, insulin secretory responses to ambient glucose concentrations generally remain unchanged, while fasting plasma insulin concentrations and day-long plasma insulin levels may decrease. MET can indirectly improve BCF by reducing the ambient level of hyperglycemia and by promoting weight loss or weight maintenance, thereby reducing glucotoxicity, lipotoxicity, and β-cell “workload.” In addition, MET often amplifies the action of other antidiabetic drugs because it provides a complementary mechanism of action. 

The seminal UKPDS study has been tracking the long-term effectiveness of treatments commonly used in the 1980s, as compared with more intensive interventions, for 15 years in a large population of patients with T2DM [5, 58-60]. Patients were initially treated with diet for 3 months, then this conventional (primarily diet) therapy or an intensive (SFU, insulin, or MET) therapy was escalated over the following 6 years in order to meet pre-established glycemic goals [58]. The intensive therapy group using SFU, MET, or insulin similarly improved glycemic control compared with the group receiving conventional diet therapy. Nonetheless, FPG, HbA_1c_, and BCF (measured as HOMA-B) continued to deteriorate progressively over time in all groups. Of additional interest, there was less weight gain and fewer episodes of hypoglycemia with MET than with SFU or insulin. The calculated HOMA-B deteriorated to a similar extent in the 37% of patients on conventional diet therapy alone (*P*<0.0001) and in the 50% of patients on SFU (*P*<0.0001). In the subgroup of obese patients treated with conventional diet or with MET, HOMA-B decreased geometrically from 60% to 33% (*P*<0.0001) or from 51% to 38% (*P*<0.0001), respectively. This ground-breaking study thus provided solid evidence of the progressive nature of the disease with glycemic control failure in all patients with T2DM receiving monotherapy that did not specifically address the underlying pancreatic β-cell defect over time. It was the first study to conclusively show that SFU had no protective effect on the β-cell. Furthermore, although there is *in vitro* evidence supporting a role for MET in improving BCF and preventing β-cell apoptosis, the UKPDS data failed to confirm any MET-mediated preservation of BCF [2]. The A Diabetes Outcome Progression Trial (ADOPT) provided further confirmatory observations to the findings reported by the UKPDS, with the additional notion that a TZD (ROSI) complements the effects of older therapeutic agents [60]. It should be emphasized that in the ADOPT study, the estimated β-cell loss was 3-fold faster in SFU-treated patients than in TZD-treated patients. Additional examples of investigations into the effects of MET and other OADs on BCF are shown in (Tables **2-4**).

#### Drugs that Stimulate Insulin Secretion Independent of Ambient Glucose Concentration 

SFUs are OADs that tend to rapidly improve glycemic control in patients with T2DM [58-60, 63-65]. Over the long term, however, SFUs lose their effectiveness and are invariably associated with a progressive rise in HbA_1c_, due to continued deterioration of BCF. These outcomes make sense when the primary mechanism of action of SFU-class drugs is considered more closely. SFUs act by stimulating pancreatic β-cells to secrete insulin by binding the SUR-1 receptor, a receptor that is disconnected and downstream from the glucose-mediated insulin secretion pathway. The insulin secretagogue mechanism behind SFUs is also independent of prevailing circulating insulin levels. There is no evidence that these agents have any beneficial effects on insulin biosynthesis, proinsulin processing, or insulin exocytosis in pancreatic β-cells. Actually, chronic use of SFUs may exhaust the pancreatic β-cell hormonal reserve and further deteriorate BCF over time. These deleterious effects are secondary to the direct, and continual, maximum stimulation exerted by drug-binding to the SUR-1 β-cell membrane receptor. Also, indirectly, SFUs do not alleviate any of the known cytotoxic factors that contribute to the accelerated disease course and progression. Hypoglycemia tends to occur much more frequently with the use of SFUs than with MET and other non-secretagogue agents in the treatment of diabetes. For these reasons, the various estimates of BCF in SFU-treated patients with T2DM provide little information with regards to the extent and progression of β-cell failure. Frequently, patients with T2DM not responding to SFU therapy are essentially at an advanced stage of the disease, and this is taken as an indication that a late stage of β-cell failure has already been reached. Unfortunately, most widely used techniques that measure BCF on a background of SFU use are misleading, because they are essentially based on plasma insulin and glucose concentrations that follow the drug intake. As a result, plasma insulin may be high and plasma glucose near normal by the time the test is performed, when β-cells have actually not undergone any biological improvement. Thus, when BCF is measured in SFU-treated patients using these methods, there is a false impression that the population of β-cells is robust and healthy when, in fact, they are “starving.” Examples of investigations into the effects of SFUs on BCF are shown in (Tables **2-4**). 

Even though the meglitinide drug class (repaglinide [RPG] and nateglinide) does not share structural characteristics with the SFU drugs, they do have a similar mechanism of action. The assessment of BCF using this method, on a background of "glitinide" therapy, falls short for the same reasons explained above [66, 67]. Due to their generally lower efficacy and shorter duration of action than most SFUs, their usage is not as prevalent and there are very few studies addressing BCF in glitinide-treated patients. Examples of investigations into the effects of meglitinides on BCF are shown in (Tables **2-4**). 

#### Insulin Sensitizers - the TZDs

In clinical trials, TZDs improved glycemic control by improving insulin sensitivity, but did not directly stimulate insulin secretion from pancreatic β-cells [2, 68, 69]. The currently available TZDs, ROSI and PIO, are peroxisome proliferator-activated receptor gamma agonists that act primarily in insulin-dependent tissues, such as liver, adipose, and skeletal muscle. These drugs are known to reduce insulin resistance in hepatic and peripheral tissues. As a consequence, insulin inhibition of endogenous glucose output at baseline and after meals are more effective, and insulin-mediated glucose metabolism is augmented. Long-term studies suggest that, after the initial decline in HbA_1c_ in patients with T2DM who are started on therapy with TZD, glycemic control is maintained for longer periods of time, presumably due to the preservation of BCF [2, 68]. 

In the ADOPT study, the effects of MET, SFU (glyburide [GLY]), and TZD (ROSI) monotherapies were directly compared in a group of newly diagnosed patients with T2DM over a median period of 4 years [62]. After 5 years of treatment, there was a cumulative incidence of monotherapy failure of 21% with MET, 34% with SFU, and 15% with ROSI. These rates correspond to risk reductions of 32% for ROSI versus MET (*P*<0.001) and 63% with ROSI versus SFU (*P*<0.001). ROSI was associated with more weight gain and edema than either MET or SFU (*P*<0.001), fewer gastrointestinal events than MET (*P*<0.001), less hypoglycemia than SFU (*P*<0.001), and more congestive heart failure than SFU (*P*<0.05). During the first 6 months, SFU reportedly increased HOMA-B more than ROSI or MET (*P*<0.05). Thereafter, however, HOMA-B declined in all treatment groups. Of note, the rate of annual decline in HOMA-B was 6.1% for SFU (*P*<0.001 vs. ROSI), 3.1% for MET (*P*=0.02 vs. ROSI), and 2.0% for ROSI. Furthermore, during the first 6 months of therapy, ROSI increased HOMA-S more than MET (*P*<0.05), followed by similar rate improvements over the next 3.5 years. At year 4, HOMA-S remained significantly increased versus MET (*P*<0.001), whereas SFU did not change HOMA-S. All of these effects occurred on a 5-year background of weight gain in the ROSI and SFU groups, and weight loss in the MET group.

In similar studies, HOMA-B displayed variable kinetics in drug-naïve patients with T2DM treated with PIO or SFU (gliclazide) during 2 years [70]. As expected, in view of the mathematical model, HOMA-B increased more with SFU than with PIO (*P*<0.05 between groups). However, this difference was substantially greater between weeks 4 and 42 (mostly due to an elevated SFU effect) than between weeks 52 and 104 when HOMA-B faded back towards baseline in the SFU group. HOMA-B in the PIO group gradually increased up to week 24, then reached a plateau at a level higher than baseline. The calculated HOMA-S decreased from baseline with SFU to a nadir by week 8 and increased with PIO to plateau by week 24 (*P*<0.0001 between groups). In a 26-week study of drug-naïve patients with T2DM, therapeutic doses of PIO given as monotherapy increased the IGI during an OGTT (*P*<0.05 vs. baseline; *P*=0.07 vs. placebo) [71]. The whole-body ISI derived from the OGTT was significantly elevated (*P*≤0.08), as was the hepatic ISI (~HOMA-S; *P*<0.05). Additional examples of investigations into the effects of TZD drugs are shown in (Tables **2-4**).

#### Drugs that Stimulate Insulin Secretion Dependent upon Ambient Glucose Concentration

Dipeptidyl peptidase-4 inhibitors (DPPIs) [72-81] and GLP-1RAs [82-86] are newer classes of anti-diabetes drugs characterized as incretin-based therapeutic agents. GLP-1 receptor stimulation leads to potentiation of insulin secretion from pancreatic β-cells under hyperglycemic conditions with simultaneous decreases in glucagon secretion from pancreatic α-cells. Stimulation of the extra-pancreatic GLP-1 receptor is also followed by inhibition of gastric emptying with reduction in intestinal motility and appetite suppression. The mechanisms of action of these two newer classes of agents are slightly different. The DPPIs directly inhibit the rapid enzymatic inactivation of endogenously secreted GLP-1 and glucose-dependent insulinotropic polypeptide (GIP), with subsequent prolonged circulating time and availability of the active peptides. The GLP-1RAs in the circulation directly bind to the receptor in the pancreatic β- and α-cells and in extra-pancreatic tissues. Once the receptor is activated, GLP-1RAs are capable of similar effects to those of the DPPIs, but with greater magnitude, and the impact in the overall hormone and substrate kinetics is notably more potent. There is an abundance of clinical data for the effects of DPPIs that include alogliptin (ALO), linagliptin (LINA), saxagliptin (SAXA), sitagliptin (SITA), and vildagliptin (VILDA). DPPIs have been shown to mildly improve glycemic control in patients with T2DM when used as monotherapy and modestly when used in combination with a variety of other treatments. The GLP-1RAs with substantial clinical trial data include exenatide (twice daily [ExBID] and once weekly [ExQW]), LIRA, and lixisenatide (LIXI). GLP-1RAs can essentially restore glycemic control in patients with T2DM when used as monotherapy and in combination with a variety of other treatments. Notably, the GLP-1RA drug class is the only current anti-diabetes category associated with progressive and sustained weight loss. In addition, GLP-1RAs slow gastric emptying to ameliorate PPG excursions [87, 88]. Taken together, these mechanisms of action, plus the amplification effect on glucose-dependent insulin secretion and suppression of glucagon release, have raised the possibility of a substantial improvement in islet cell function over time in patients with T2DM managed with incretin-based therapies. Clinical studies have provided sufficient evidence supporting the ability to enhance BCF, with generally little or no effect on basal insulin sensitivity/resistance. 

##### DPPIs

ALO, SITA, VILDA, SAXA, and other DPPIs have produced augmentation of pancreatic β-cell mass in nonclinical models of insulin resistance and T2DM *via* stimulation of β-cell neogenesis, stimulation of β-cell proliferation, and suppression of β-cell apoptosis [89-94]. In clinical trials ranging from 6 weeks to 1 year, DPPI drugs have demonstrated weak improvements in FPG concentrations and modest postprandial decreases, accompanied by favorable changes in fasting and postprandial plasma insulin responses, HbA_1c_, and some measures of BCF (*e.g.,* HOMA-B, IGI, AUC_insulin/glucose_) [2, 94]. The fact that DPPIs also inhibit glucagon secretion, combined with the concomitant rise in plasma insulin levels, promotes an effective suppression in basal EGP. However, no clinically relevant effects on basal insulin resistance have been reported (HOMA-IR/HOMA-S) [95-100]. 

The effects of DPPIs on measures of fasting homeostasis in patients with T2DM can be exemplified by four reports derived from 24-week clinical trials studying SITA or SAXA [95, 97-99]. In the first study, SITA was investigated in patients with T2DM inadequately controlled on SFU±MET [95]. Significant increases were observed in fasting plasma insulin concentration (*P*<0.001) and the calculated HOMA-B, from a baseline value of 50.7% to 61.4% at week 24 (+12.0% vs. placebo; *P*<0.05). There were no significant changes in fasting proinsulin concentration or the ratio of proinsulin-to-insulin. In another clinical study, SAXA monotherapy in drug-naïve patients improved HOMA-B from a mean baseline of 65.5% to 81.0% at week 24, a significant improvement compared with both baseline and placebo (*P*<0.05) [99]. In the third clinical study, drug-naïve patients received either SAXA monotherapy, MET monotherapy, or the combination (MET+SAXA) [98]. HOMA-B improved in all groups, with significantly greater improvement in the MET+SAXA combination compared with either monotherapy (*P*<0.001). Finally, on a background of TZD (primarily PIO) therapy, SAXA again improved HOMA-B versus placebo [97]. Of note, SAXA-treated patients who had below-median baseline HOMA-B values had greater reductions in placebo-subtracted HbA_1c_ change from baseline values. This subgroup also had higher mean HbA_1c_ at baseline, indicating that this represented a subpopulation with worse glycemic control (*i.e.,* presumably greater extent of β-cell failure).

It should be mentioned that these results further exemplify some of the critical limitations inherent in the use of the HOMA and proinsulin-to-insulin ratio measurements as methods for assessing the effects of various therapies on BCF. These data highlight the absolute lack of agreement between these two methodologies. As a consequence of these discrepancies, and due to methodological difficulties, it becomes nearly impossible to derive any practical conclusions of clinical significance about BCF changes in patients with T2DM treated with either DPPIs or GLP-1RAs, based solely on data obtained from these two methods. This highlights the need for the application of more sophisticated and reproducible methods to explore the dynamic relations between glucose and insulin in patients with diabetes, especially in those managed with incretin-based therapies.

The effects of DPPIs on measures of the relationships among whole-body glucose metabolism, tissue insulin resistance, and islet-cell function after a nutrient load can be exemplified by a report from Foley *et al.* [101]. In drug-naïve patients with T2DM, 52 weeks of treatment with VILDA or placebo, followed by a 12-week washout period, was studied using a hyperglycemic clamp with arginine stimulation to assess pancreatic islet-cell function and by a euglycemic-hyperinsulinemic clamp to determine the degree of tissue insulin resistance in each individual. At the end of 1 year of VILDA treatment there were improvements in the first and second phase insulin responses to glucose (between-group adjusted mean difference: +0.77±0.38 [*P*=0.047] and +9.89±3.19 nmol/L*min [*P*=0.003], respectively). In addition, VILDA monotherapy increased the acute plasma insulin response to arginine (AIR_arg_) by +5.0±1.8 nmol/L*min, versus a decrease of -0.8±1.8 nmol/L*min with placebo (*P*=0.03 between groups). No effect on insulin sensitivity was observed, as the *M* values were increased by +0.77±1.9 mg*kg^−1^*min^−1^ and +0.55±1.72 mg*kg^−1^*min^−1^, for VILDA and placebo, respectively. Of interest, there was a trend for a VILDA-induced increase in the calculated DI (AIR_arg_*M value) by +49.6±15.0 nmol*mg*L^-1^*kg^-1^, but this did not reach statistical significance. Unfortunately, all between-group differences had disappeared by the end of the 12-week washout period, suggesting that there was no permanent improvement of BCF associated with VILDA therapy. This type of meager response to DPPI therapy was confirmed in another clinical study in drug-naïve patients with T2DM using SAXA monotherapy versus placebo for 24 weeks, followed by open-label SAXA for an additional 24 weeks [96]. During the placebo-controlled treatment period in this study, SAXA 10 mg once daily (QD) reduced plasma glucose concentration from baseline at all time points during a standard OGTT. At 120-min post-glucose load, the adjusted-mean change was -3.0 mmol/L versus -0.3 mmol/L in the placebo group (*P*<0.0001). Furthermore, SAXA monotherapy was accompanied by an increase in postprandial insulin and C-peptide AUC, with suppression of postprandial glucagon AUC, relative to placebo. A small improvement in insulin sensitivity in the postprandial (oral glucose insulin sensitivity index [OGIS]), but not in the fasting (HOMA-IR), state was also demonstrated. However, because the MI and IGI did not change, no clinically meaningful improvement in whole-body insulin resistance can be inferred from these data. At the end of the open-label extension with SAXA 10 mg QD, post-challenge plasma glucose at the 120-min time point during the OGTT had decreased by -3.7 mmol/L from baseline (*P*<0.05). Total PPG AUC was also significantly reduced (*P*<0.05). Finally, on a background of TZD (primarily PIO), SAXA for 24 weeks decreased glucose concentration at all time points during an OGTT [94]. At the 120-min time point during the OGTT, PPG change from baseline was -4 mmol/L for SAXA versus -1 mmol/L for placebo. Greater reductions in 120-min PPG were observed with SAXA than with placebo (*P*<0.0001). The IGI also increased from baseline with SAXA, but decreased with placebo.

An example of using a comprehensive mathematical modeling approach to understand the physiological changes in drug-naïve patients with T2DM during DPPI treatment was reported by Mari *et al.* [102]. Four weeks of VILDA therapy improved BCF by significantly increasing insulin secretory rate (calculated from the dose-response curves) at a reference glucose concentration of 7 mmol/L (*P*=0.002) in this placebo-controlled study. According to this model, the slope of the β-cell dose response, the derivative component, and the potentiation factor were not affected.

 In summary, improvements in HOMA-B and some dynamic measures of BCF have consistently been demonstrated across the DPPI drug class. However, these improvements were not sustained after drug withdrawal. There are no apparent differences among the DPPI drugs currently available. Further examples can be found in (Tables **2-4**) and the review article by Van Genugten *et al.* [94].

##### GLP-1RAs

Numerous nonclinical experimental models of T2DM and insulin resistance have been studied in an effort to assess the effects of GLP-1RAs on islet-cell morphology and on BCF, in conjunction with measurements of the degree of insulin resistance [86, 103, 104]. Gedulin *et al*. [105] used the nondiabetic obese Zucker rat model of insulin resistance to isolate the effects attributable to improved glycemic control from those attributable to reductions in food intake and body weight. During the disease process in this animal model, values of HbA_1c_ and fasting plasma insulin concentration rose progressively, primarily because this rat strain is genetically predisposed to hyperphagia, obesity, and insulin resistance. This study also examined the effects of exenatide on β-cell mass dissociated from the changes in glycemia and insulin sensitivity. Insulin sensitivity, estimated as the glucose infusion rate required to maintain euglycemia at 180 min divided by the steady-state plasma insulin concentration during the euglycemic-hyperinsulinemic clamp, was 224% higher in *ad libitum*-fed exenatide-treated rats than in the *ad libitum*-fed control rats (*P*<0.001). Insulin sensitivity was also 61% higher in *ad libitum*-fed exenatide-treated rats than in pair-fed control rats (*P*<0.003), despite comparable HbA_1c_, fasting glucose, fasting insulin, total cholesterol, HDL-C, and food consumption. In *ad libitum*-fed exenatide-treated rats, the index of “β-cell mass x insulin sensitivity” was 63% greater than in the pair-fed control rats (*P*<0.05). Thus, the study concluded that exenatide infusion increased β-cell mass to a greater extent than would be expected in animals with comparable degrees of insulin resistance. These data suggest that exenatide may exert a direct trophic effect on pancreatic β-cell mass, presumably by stimulating islet neogenesis, independent of any improvements in metabolic parameters. 

Various reports have also demonstrated augmentation of pancreatic β-cell mass with continued exposure to a GLP-1RA, primarily in animal models of insulin resistance and T2DM. These observations have been interpreted as potential evidence that GLP-1RAs may promote β-cell neogenesis and proliferation, concurrently with suppression of β-cell apoptosis [88, 103, 104]. In similar experimental models, GLP-1RAs have been shown to up-regulate expression of the genes responsible for augmented insulin bio-synthesis and secretion. Because the development of the endocrine pancreas is under a multifactorial control [106], it has been proposed that GLP-1RAs may induce early differentiation of pancreatic non-β-cells, including pancreatic ductal epithelial cells, acinar cells, and nestin-positive periductal cells (potentially ‘‘functional’’ islet stem cells) into insulin-producing β-cells [107]. However, these promising results have only been reported in *in vitro* and *in vivo *experimental animal studies, and definitive data in humans are still lacking. In another example, the demonstration of an increased expression of the transcription factor PDX-1/IDX-1, a factor essential for the development and regeneration of the endocrine pancreas, suggested that this may be one important intracellular mechanism by which GLP-1RAs can enhance β-cell mass. GLP-1RAs can also induce AR42J pancreatic tumor cells, a cell line that is negative for islet hormones and their respective mRNAs, but can proliferate and differentiate into cells with positive immunohistochemical staining for insulin. Nonetheless, a functional correlation of these morphological and developmental findings in islet cells has not yet been established in patients with T2DM.

The effects of GLP-1RAs on measures of fasting homeostasis (HOMA) can be exemplified by results obtained in a clinical trial of ExBID monotherapy for 24 weeks in treatment-naïve T2DM patients where HOMA-B improved by 28%, compared with only 6% in the placebo control group, starting with baseline values of 45.3%-50.0% across groups (*P*=0.01) [108]. In clinical trials, under steady basal conditions of plasma glucose and insulin, both exenatide (BID, QW) and LIRA improved HOMA-B and the ratio of proinsulin-to-insulin [109-121]. In contrast, however, HOMA-IR/HOMA-S values were not affected by ExBID, LIRA QD, or ExQW in many other similar clinical studies [110, 112, 115, 117-125]. A few atypical reports of mild changes in these weak measures of BCF have been published, but these are probably not clinically meaningful [111, 116, 126]. 

In clinical pharmacology studies conducted in T2DM patients, exenatide therapy was shown to modulate serum insulin in a manner dependent upon both the exenatide dose and the ambient glucose concentration [127-129]. ExBID for 28 days increased HOMA-B by 50%-100% over baseline versus no change in the placebo group on a background of MET and/or SFU [109]. These results were substantiated in six other clinical trials in patients with T2DM treated with ExBID, on the same background therapy, over periods ranging from 0.5 to 3 years [110, 111, 121, 123, 126, 130, 131]. In a 6-month study, ExBID increased mean HOMA-B from a baseline of 33% to 43% (*P*=0.02) [110] and, in one of the pivotal phase 3 clinical trials on a background of SFU therapy, 30 weeks of ExBID reduced the ratio of proinsulin-to-insulin by -0.13 from a baseline of 0.66 (*P*=0.001 vs. placebo) [130]. In a second pivotal trial, on a background of MET therapy, 30 weeks of ExBID also significantly reduced the ratio of proinsulin-to-insulin (*P*<0.001 vs. placebo) [131]. Furthermore, in a 2-year study, mean HOMA-B improved by 49% versus baseline (*P*<0.01) [111], with further improvement noted after 3 years of exenatide therapy (mean HOMA-B at baseline: 52.4%; 3-year: 70.1%; *P*<0.0001) [123]. In a different clinical trial, 1 year of ExBID improved HOMA-B from a mean of 48.8% at baseline to 67.6% (*P*<0.001) on a MET+SFU background therapy [121] and another 1-year study yielded comparable results on a background of MET monotherapy [126]. In the latter study, patients with T2DM on a controlled-energy diet (~600 kcal daily deficit) and increased physical activity displayed increases in HOMA-B with ExBID from a baseline mean of 57.8% to 69.5% (*P*<0.01 vs. baseline; *P*<0.05 vs. SFU). 

Similar results have been reported in clinical trials in which patients with T2DM had a concomitant background therapy that included a TZD as monotherapy or in combination (TZD±MET, TZD±SFU) [122, 124, 125]. In two of these studies, HOMA-B improved from baseline after 16 or 26 weeks of exenatide treatment [122, 124]. In the third study, HOMA-B improved equally in the exenatide and placebo groups after 24 weeks of treatment [125]. Of note, this was the only GLP-1RA study in a T2DM population with healthy body weight (mean BMI, 25.5 kg/m^2^). 

LIRA QD at three different doses for 14 weeks improved mean HOMA-B by 75%-134% versus placebo (*P*<0.0001), and the median ratio of proinsulin-to-insulin also decreased versus the placebo group (*P*≤0.02) [112]. In another study, a direct comparison of LIRA QD for 26 weeks improved HOMA-B by 32% versus a 3% improvement with ExBID (*P*<0.0001 between groups) in a group of patients with T2DM receiving adjunctive treatment with either MET, SFU, or MET+SFU [113]. In this study, the mean ratio of proinsulin-to-insulin was unchanged by either treatment. In a separate 26-week study, LIRA QD was compared to insulin glargine (GLARG) on a background of MET+SFU in patients with T2DM, and the mean ratio of proinsulin-to-C-peptide decreased significantly compared with GLARG therapy (*P*=0.0019) [114]. When LIRA QD was compared to placebo injections in T2DM patients taking MET+ROSI for a period of 26 weeks, HOMA-B improved from a baseline of 34%-37% to 61%-64% (*P*<0.0001), whereas the mean ratio of proinsulin-to-insulin versus placebo decreased significantly (*P*<0.05) [115]. In contrast, treatment with LIRA QD or SFU (glimepiride [GLIM]) monotherapy for 52 weeks did not demonstrate any significant change in either HOMA-B or in the ratio of proinsulin-to-insulin between treatment groups [116]. When comparing LIRA QD to either a TZD (ROSI) or a placebo therapy on a background of SFU (GLIM), the mean ratio of proinsulin-to-insulin decreased more with LIRA than with TZD or placebo (*P*≤0.02) [117], though LIRA improved HOMA-B more than TZD (*P*=0.01). 

In drug-naïve patients with T2DM, the difference between the HOMA-B value at the 26-week endpoint and the baseline HOMA-B value was significantly greater with ExQW monotherapy (+1.8±0.06; *P*<0.001) versus monotherapy with either MET (+1.4±0.04), PIO (+1.3±0.05), or SITA (+1.3±0.04) [119]. In addition, on a background therapy of MET±SFU, treatment with ExQW was accompanied by a greater increase in HOMA-B than was treatment with GLARG, with an ExQW-to-GLARG 26-week change ratio of 1.26 (*P*<0.05) [118]. Significant reductions in HOMA-B values were also demonstrated after 3 years of ExQW treatment in patients with T2DM on a background therapy of MET, SFU, TZD, or any combination of two of these drugs [120]. 

The effects of GLP-1RAs on measures of the dynamic relationship between glucose metabolism and BCF after a nutrient load can be exemplified by reports from Bunck and colleagues [132, 133]. At the end of 1 year of treatment, BCF was assessed in MET-treated patients with T2DM given ExBID or GLARG titrated to reduce HbA_1c_ to approximately 6.8% [132]. Under a standard hyperglycemic clamp with the additional arginine stimulation performed at the end of the procedure, both first- (0-10 min) and second-phase (10-80 min) insulin secretions were increased by 1.5- and 2.9-fold, respectively, with ExBID versus GLARG (*P*<0.0001). Arginine-stimulated plasma C-peptide concentrations increased 3.2-fold when compared with baseline after exenatide therapy versus only 1.3-fold with GLARG therapy (*P*<0.0001 between groups). However, these impressive results were not sustained, and measures of BCF had returned to pretreatment levels in both groups 4 weeks after drug discontinuation. Of interest, in a follow-up to this observation, after 3 years of treatment and 4 weeks off-drug, first-phase insulin secretion adjusted for insulin sensitivity (DI) demonstrated a sustained improvement in the exenatide group, whereas GLARG slightly reduced this measure of BCF (*P*=0.028 between groups) [133]. Moreover, in a separate measurement, at the end of 52 weeks neither ExBID nor GLARG changed the whole-body insulin sensitivity (the *M* value), although 4 weeks after drug discontinuation insulin sensitivity remained improved in the exenatide group (*M* value increased by 39%; *P*=0.006), but not in the GLARG group. The calculated DI measured during the second-phase (10-80 min) insulin secretion during the hyperglycemic clamp was not significantly different between groups.

In an independent and separate 14-week study, LIRA improved first-phase insulin response (AUC_0-10 min_) during an IVGTT by 103% compared with placebo (*P*=0.05), but did not achieve as large a response as was observed in healthy, untreated, non-diabetic volunteers [134]. Similar results were reported for the incremental AUC_0-10 min_ and some, but not all, doses of LIRA also improved the second-phase insulin response (AUC_19-40 min_) compared with placebo. Similar to the study mentioned above for exenatide [132], during a standard hyperglycemic clamp procedure with a later arginine-stimulation test, LIRA improved first- and second-phase insulin response by 118% and 79%, respectively, versus placebo (*P*<0.05) [134]. One additional interesting observation comes from a crossover study in patients with T2DM and inadequate glycemic control receiving MET who were randomized to treatment with either ExBID or SITA for 2 weeks on each arm [135]. In this study, during an MTT, the group of patients receiving exenatide showed a significant improvement in acute ISRs (ISR_0-30 min_) and in the IGI, which was greater than that observed in the SITA-treated group (*P*≤0.02). In a similar study using a two-way crossover design, patients with T2DM were randomized to a single subcutaneous injection of LIXI or placebo [86]. Two hours later, patients received an intravenous bolus of glucose (IVGTT). LIXI was noted to increase first-phase insulin response by more than 6-fold and to restore second-phase insulin secretion by ~3-fold versus placebo. Further examples can be found in (Tables **2-4**).

An example of using a comprehensive mathematical modeling approach to understand the physiological changes during GLP-1RA treatment is provided in a report by Mari *et al.* [37]. In this study, data from patients with T2DM treated with ExBID or placebo on a background of MET±SFU for 30 weeks were analyzed. ExBID during an MTT reduced PPG excursions, unlike placebo, and modeling predicted an upward shift of the β-cell dose response. The model predicted that ISRs at a given reference glucose concentration would increase by 72% from baseline with ExBID treatment, compared with a 21% decrease with placebo (*P*=0.02 between groups). At week 30, the 2-hour post-meal-to-basal potentiating factor ratio was estimated to have increased to 1.53±0.10, compared with only 1.15±0.06 in the placebo group (*P*=0.01 between groups). Thus, the mathematical model predicted that ExBID treatment would be associated with an upward shift of the β-cell dose response, with an enhanced potentiating of insulin secretion.

In summary, because the dynamic tests are more reliable methods of assessing changes in BCF than are the homeostatic tests, it seems reasonable to assume that GLP-1RAs are more likely capable of restoring physiological β-cell insulin secretion patterns in all stages of glucose intolerance, as compared with insulin and other antidiabetes therapies. For the most part, however, any perceived improvements in BCF while patients are in treatment are not sustained upon discontinuation of the drugs. Although these effects may be apparently lost immediately after discontinuation of the GLP-1RA therapy, there seems to be a long-term imprint effect on the first-phase insulin secretion, which is worth pursuing in all patients with T2DM who can tolerate, and are good candidates for, GLP-1RA therapy. Finally, improvements in both homeostatic and dynamic measures of BCF have been consistently demonstrated across the GLP-1RA drug class, but whether these have any impact on the natural history of the disease, which translates into clinical benefits to patients with T2DM, remains to be determined. 

## SUMMARY AND CONCLUSION

**Fig. (6):**
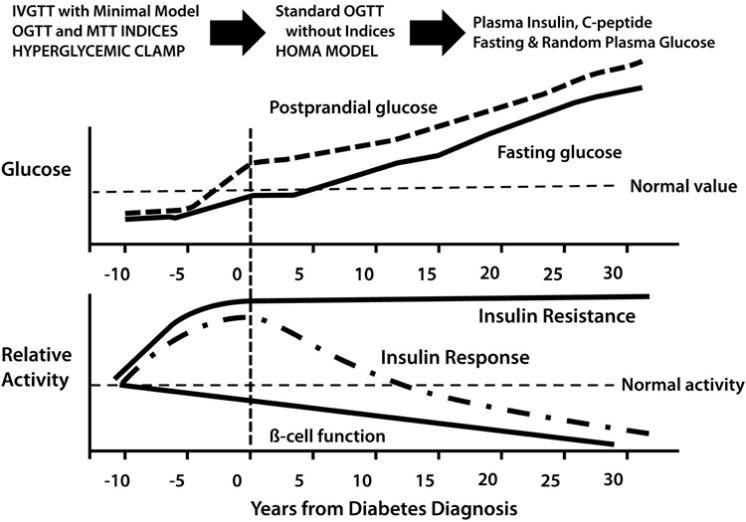
Schematic representation of the natural progression of T2DM highlighting the need for BCF measurements to devise optimal treatment strategies. Top panel: Shows the initial subclinical elevation of the postprandial glucose followed by overt fasting hyperglycemia. The disease process, however, begins 5-10 years prior to the actual diagnosis. Lower panel: Diabetes starts with an early development of severe insulin resistance, both hepatic and peripheral. This insulin resistance is fully compensated by a proportionate increase in pancreatic β-cell insulin oversecretion. This balance maintains normoglycemia, but already reflects some degree of β-cell dysfunction. By the time insulin resistance reaches a near-maximum, clinically relevant hyperglycemia has manifested. This is coincident with further deterioration of the BCF, characterized by a progressive failure to secrete sufficient insulin to maintain normoglycemia. The pathogenesis of T2DM provides opportunities for therapeutic interventions to slow or prevent the appearance of frank hyperglycemia. The early and accurate diagnosis of the degree of β-cell dysfunction is critical to enable these interventions. Currently, the hyperglycemic clamp procedure with a C-peptide deconvolution analysis, the IVGTT minimal model with calculation of the disposition index, and the insulin secretion indices derived from a MTT represent superior methods of assessing BCF. Applying the HOMA model and/or the standard OGTT, although more convenient and easier to implement, are less sensitive methods for the detection of β-cell dysfunction as the disease progresses. In the later stages of the disease, the determination of plasma insulin, C-peptide, fasting and random plasma glucose levels, which are routine measurements, will indicate advanced β-cell failure. BCF, β-cell function; HOMA, homeostasis model assessment; IVGTT, intravenous glucose tolerance test; MTT, meal tolerance test; OGTT, oral glucose tolerance test; T2DM, type 2 diabetes mellitus. Modified from [185].

The pathophysiology of T2DM is characterized by progressive β-cell failure on the background of insulin resistance (Fig. **6**). Based on extensive review of the literature, it appears that the development of an optimal treatment strategy for each patient with T2DM, as well as for those with prediabetes, will require an accurate early diagnostic assessment of the degree of residual BCF within the context of each individual’s insulin resistance. In this review, we focused on the most widely used methods for the evaluation of BCF and their clinical applications.

HOMA was developed to reflect the balance between hepatic glucose production and β-cell insulin secretion during the basal homeostatic state. It is calculated using the steady-state fasting glucose and insulin concentrations to concomitantly estimate the degrees of β-cell deficiency and the target-tissue sensitivity to insulin. In order to examine the dynamic relationship between these parameters, one can utilize more complex tests such as the IVGTT, OGTT, MTT with indices, and the hyperglycemic clamp. The IVGTT with minimal model analysis quantifies first-phase (acute) insulin secretion and second-phase (more prolonged) insulin secretion after an intravenous bolus of glucose. The model assumes that glucose acts to increase its own utilization and to retard its endogenous production in direct proportion to plasma glucose concentration. Insulin is assumed to synergize with these glucose effects and promote further glucose disappearance from plasma in a concentration-dependent manner. The IVGTT is amenable for use in a routine clinical setting with minimal patient risk and yields perhaps the most accurate measurement of the first-phase insulin secretory response to a carbohydrate stimulus. The IVGTT is limited, nonetheless, because it cannot predict the variable glucose absorption rates from the gut, and thus ISRs calculated using this approach represent a theoretical and artificial response to a non-physiological intravenous glucose load. The OGTT and MTT provide means of assessing insulin secretory patterns in more physiological conditions, as these tests include the gastrointestinal incretin effect that follows oral nutrient ingestion. The primary measures of the β-cell pancreatic responses are also the determination of the magnitude of the first- (acute) and second-phase insulin secretion combined, after an oral nutrient load. Typically, these values are captured from the incremental AUC. Although the acute and prolonged phases of insulin secretion using these oral intake methods cannot be as sharply delineated as in the IVGTT, the inclusion of the glucose absorption from the gut is one important factor that contributes to the overall pancreatic insulin secretory process. Use of a two-compartment oral minimal model can provide two global indices of BCF, namely the ISR related to increased adiposity and a β-cell index that takes into account a patient's glucose tolerance state. The MTT is more difficult to standardize and more cumbersome to administer than the OGTT, but it more closely reproduces the normal physiologic state after consumption of a mixed meal, and thus provides data of greater clinical and nutritional relevance.

The hyperglycemic clamp is a highly reproducible method of assessing β-cell sensitivity to glucose under maximal stimulatory conditions. This technique enables accurate and reproducible follow-up estimates of BCF in any condition where the insulin response either improves or deteriorates as a result of changes in plasma glucose levels over time. It can be applied to investigate BCF in longitudinal studies of small populations, and to compare BCF before and after therapeutic interventions.

The gold-standard method for quantitating the degree of insulin resistance (*i.e.,* the euglycemic-hyperinsulinemic clamp procedure) was discussed to provide a background for the correct assessment of insulin secretion in patients with prediabetes and T2DM under various therapies. Finally, the Mari/Ferrannini mathematical model represents an approach that derives data from some of the discussed dynamic tests used in evaluating BCF and can be used to develop population-wide conclusions concerning the overall status of BCF and glucose tolerance. The model incorporates most factors known to be involved in the regulation of the pancreatic β-cell response to glucose, allowing for theoretical explorations of the dynamic relationships between glucose and insulin concentrations. 

We also reviewed different therapeutic interventions commonly used in patients with prediabetes or T2DM, with an emphasis on β-cell preservation. Dietary manipulations with regular physical activity, followed by weight loss and better glycemic control, are often accompanied by attenuation of the rate of progression β-cell loss, thus slowing or halting the advancement of the disease. In addition, they are usually associated with simultaneous improvements in hypertension, dyslipidemia, and other CVD risk markers. Although lifestyle interventions are considered first-line therapy for prediabetes and for patients with newly diagnosed T2DM, nearly all of the weight loss resulting from lifestyle modifications is regained within 5 years. Therefore, more creative approaches become necessary in order to maintain adequate body weight and glycemic control. Most experts recommend the use of MET as first-line pharmacotherapy. It may help to slow the conversion rate from prediabetes to overt hyperglycemia. However, although MET inhibits hepatic glucose production and mildly affects peripheral insulin sensitivity, it does not directly stimulate insulin secretion from β-cells. Thus, the use of MET alone will not suffice to prevent disease progression to any great extent. 

The SFUs and meglitinides are also used as first- and second-line antihyperglycemic agents, and are known to directly stimulate pancreatic β-cell insulin secretion, independent of ambient glucose or insulin concentrations. As a consequence, the use of these agents is more frequently associated with hypoglycemia than with any other antidiabetic pharmacotherapy, except for insulin. Due to their mechanisms of action, most methods measuring BCF typically yield misleading results because, in the presence of SFU or meglitinide therapy, variations in plasma glucose and insulin concentrations may appear beneficial. In fact, the pancreatic β-cells are depleted and their ability to synthesize and store proinsulin and insulin continues to rapidly deteriorate. TZDs improve glycemic control primarily by improving insulin sensitivity in the liver and peripheral tissues, but they do not directly stimulate pancreatic insulin secretion. Nonetheless, there is a lasting effect on the preservation of residual β-cell insulin secretory capacity that seems to be indirect and is believed to play a role in halting disease progression. 

The DPPIs and GLP-1RAs potentiate insulin secretion from pancreatic β-cells under hyperglycemic conditions and decrease glucagon secretion from pancreatic α-cells. GLP-1RAs also slow gastric emptying and nutrient absorption from the intestinal tract. Notably, the GLP-1RA agents are the only anti-diabetes drug class associated with progressive and sustained weight loss. In nonclinical models (cultured cells and experimental animal models), DPPIs and GLP-1RAs have been demonstrated to augment pancreatic β-cell mass under conditions of insulin resistance or overt T2DM *via* stimulation of β-cell neogenesis and proliferation, and suppression of β-cell apoptosis. Incretin-based therapy has been demonstrated to be consistently accompanied by significant improvements in pancreatic islet-cell function using both homeostatic and dynamic BCF measurements in humans. Whether these agents can sustain their beneficial effects and delay the progressive natural course of T2DM over longer periods of time remains unknown. 

In conclusion, we believe that early and more accurate BCF assessment can be achieved with the use of methods that provide an index of insulin secretion that takes into consideration the degree of insulin resistance in each individual. The DI comes close to this goal, as does the insulin secretion/insulin resistance index. The insulin secretion estimates derived from the C-peptide deconvolution curve may provide similar results. These indices can be derived from dynamic tests such as the OGTT and MTT with indices and from the IVGTT minimal model. To acquire an enhanced and reliable index of insulin resistance, the euglycemic-hyperinsulinemic clamp procedure may be performed in small groups of subjects. These indices are very sensitive and reproducible over time and can be useful tools in the follow-up of β-cell failure following therapy. Slowing β-cell failure is an important part of the overall treatment of diabetes, and any effective therapeutic strategy should be able to slow the progression in the initial stages of the disease (prediabetic state). This is critical because we know that the disease usually begins 5-10 years prior to the actual clinical diagnosis. Applying the HOMA model, and/or a standard OGTT, may be more convenient and relatively simple, but the results are less sensitive for the detection of changes in BCF and should not be used as the only tools for the assessment of pancreatic reserve. The estimation of BCF in the advanced stages of the disease can be attained with the use of routine determination of plasma insulin, C-peptide, and glucose concentration. However, there is a good chance that β-cell failure has reached an irreversible phase and it is too late to act, since the ultimate goal is to achieve a meaningful retardation of the appearance of hyperglycemia and the development of frank diabetes and its complications. Whether better knowledge of the degree and rate of deterioration of BCF early in the disease process provides an additional tool to help manage diabetes and prediabetes remains to be determined.

## Figures and Tables

**Table 1.** Methods Commonly Used for Measuring BCF and Supporting Procedures for Quantitation of Insulin Sensitivity.

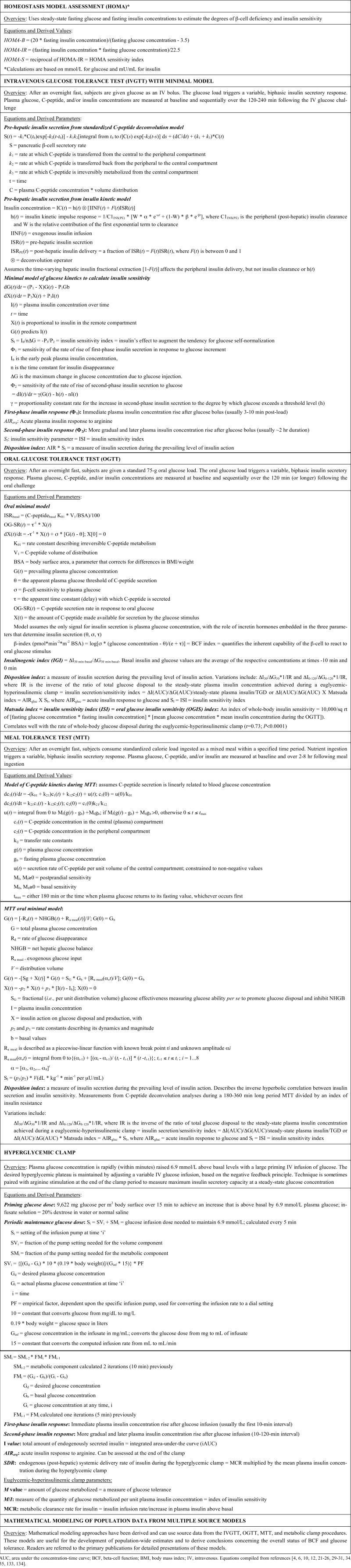

**Table:** 

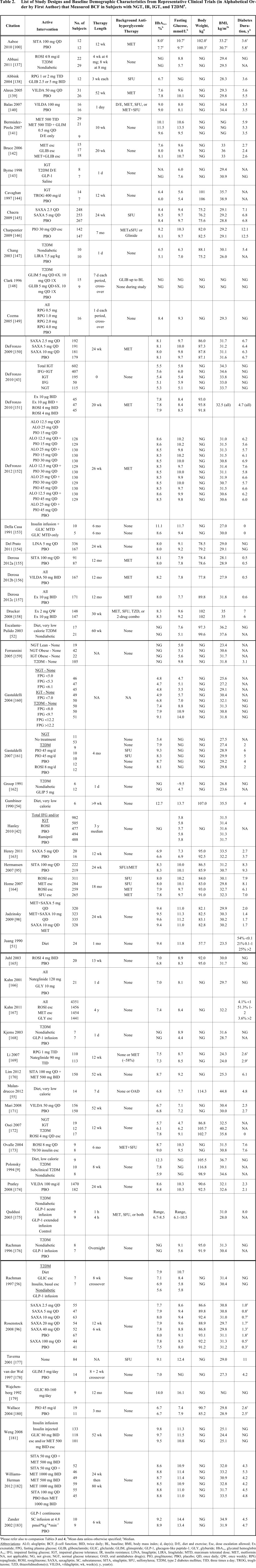

**Table 3.** Key Findings from Selected Clinical Trials that Measured BCF in Subjects with NGT, IR, IGT, or T2DM^a^.

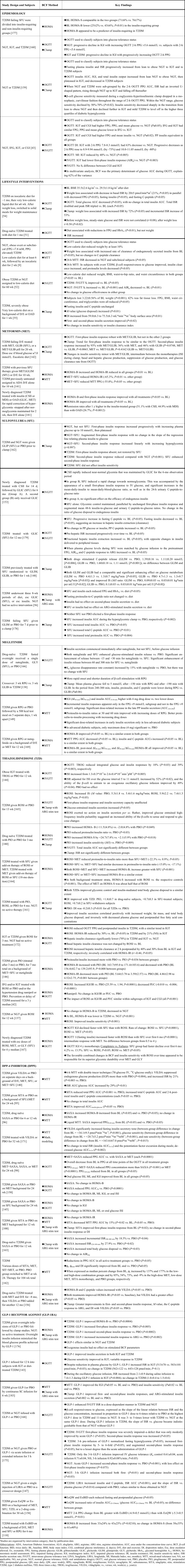

a Studies are organized by intervention category, then by publication date.

Abbreviations: ADA, American Diabetes Association; ALO, alogliptin; ARG, arginine; ARG stim, arginine stimulation; AUC, area under the concentration-time curve; BCF, β-cell function; BID, twice daily; BL, baseline; BMI, body mass index; CGI, combined glucose intolerance; d, day(s); D/E, diet and exercise; DI, disposition index; Esc, dose escalation allowed; Ex, exenatide; FPG, fasting plasma glucose; GLIB, glibenclamide; GLIC, gliclazide; GLIM, glimepiride; GLY, glyburide; HbA_1c_, glycated hemoglobin A_1c_; HOMA, homeostasis model assessment; IFG, impaired fasting glucose; IGI, insulinogenic index; IGT, impaired glucose tolerance; IR, insulin resistance; ISI, insulin sensitivity index; ISR, insulin secretion rate; IVGTT, intravenous glucose tolerance test; LINA, linagliptin; LIRA, liraglutide; MI, Matsuda Index; mo, month(s); MTT, mixed-meal tolerance test; NA, not applicable; NG, not given; NGT, normal glucose tolerance; OAD, oral antidiabetes drug(s); OGTT, oral glucose tolerance test; PBO, placebo; PIO, pioglitazone; PP, postprandial; PPG, postprandial glucose; QD, once daily; QW, once weekly; RPG, repaglinide; ROSI, rosiglitazone; SAXA, saxagliptin; SC, subcutaneous; SITA, sitagliptin; stim, stimulation; T2DM, type 2 diabetes mellitus; TID; three times a day; TROG, troglitazone; VILDA, vildagliptin; vs., versus; wk, week(s); y, year(s).

**Table 4.** Selected Applications of BCF Tests that Include Physiological Assessments of Hormone and Glucose Changes in Subjects with Different Degrees of Glucose Intolerance and Under Different Treatment Regimens.

Subjects and Glucose Status	Treatment	Key Findings
**FASTING HOMEOSTASIS BETWEEN GLUCOSE AND INSULIN (HOMA)**		
T2DM [152]	ALO±PIO on MET background for 26 wk	Proinsulin/insulin and HOMA-B improved more with ALO+PIO than with PIO alone (*P*<0.01) ALO had no additional effect on HOMA-IR over PIO alone
**DYNAMIC RELATIONSHIP BETWEEN GLUCOSE AND INSULIN AFTER NUTRIENT LOAD**		
T2DM, drug naïve [170]	SITA+MET for 52 wk	HOMA-B increased from 50.3±33.5 to 75.1±32.8 (*P*<0.01) OGTT IGI increased from 11.3±1.3 to 35.0±6.3 (*P*<0.01) Multivariate regression analysis: HbA_1c_ reduction significantly associated with high baseline HbA_1c_, low IGI, and short duration of diabetes after adjusting for age, sex, BMI, blood pressure, triglycerides, creatinine, hsCRP, glucagon, C-peptide, HOMA-B, and HOMA-IR
T2DM [173]	ROSI or 70/30 insulin esc for 6 mo	Proinsulin-to-insulin ratio decreased with ROSI by 36% (*P*=0.03); no change with insulin IVGTT AIR_g_ improved with ROSI (*P*<0.001) IVGTT S_I_ (ISI) improved by 92.3% with ROSI; no improvement with insulin ROSI: IVGTT DI increased from 0.18 at BL to 4.18 (*P*=0.02); no change with insulin
T2DM [154]	LINA or PBO for 24 wk	Tests: HOMA-B, HOMA-IR, MTT, DI LINA improved HOMA-B (*P*=0*.*049 vs. PBO) and proinsulin/insulin ratio (*P*=0*.*025 vs. PBO); no change in HOMA-IR LINA: MTT 2-h PPG reduction from BL of -3.2±0.7 mmol/L (*P*<0*.*0001 vs. PBO) LINA: MTT DI improved (*P*=0*.*0005 vs. PBO)
T2DM [139]	VILDA or PBO on MET background for 52 wk	MTT insulin secretion increased with VILDA; reduced with PBO (*P*=0.018 between groups) MTT insulin sensitivity improved with VILDA; no change with PBO (*P*=0.036 between groups) Adaptation index (pre-hepatic insulin secretion x oral glucose insulin sensitivity) increased with VILDA; decreased with PBO (*P*=0.04) Change in adaptation index correlated with change in HbA_1c_ (r=-0.39;* P*=0.004)
T2DM, drug naïve [174]	VILDA or PBO for 24 wk	VILDA increased HOMA-B vs. BL (+10.3±1.5) and vs. PBO (*P*=0.01); decreased proinsulin-to-insulin ratio vs. BL (-0.05±0.01) and PBO (*P*<0.001) VILDA improved all MTT-derived parameters (*P*<0.05 vs. BL) VILDA improved MTT ISR/G (*P*<0.001) and IGI vs. PBO (*P*=0.045)
**HYPERGLYCEMIC CLAMP WITH/WITHOUT ARGININE STIMULATION**		
T2DM, drug naïve [155]	SITA or PBO on MET background for 12 mo	SITA: Increased in HOMA-B and reduced HOMA-IR more than PBO (*P*<0.05) SITA, but not PBO, decreased proinsulin-to-insulin ratio (*P*<0.01 vs. BL) Clamp: SITA had greater improvements in first- and second-phase insulin response, *M* value, AIR_arg_, and DI (*P*<0.05 vs. PBO) Regression analysis: Correlation between HbA_1c_ reduction and *M* value increase (*P*<0.034), greater first-phase insulin response (*P*<0.022), greater second-phase insulin response (*P*<0.025), AIR_arg_ (*P*<0.031), and DI (*P*<0.043)
T2DM, drug naïve [156]	VILDA or PBO on MET background for 12 mo	VILDA: Improved HOMA-B and HOMA-IR vs. PBO (*P*<0.05) Clamp: VILDA improved first- and second-phase insulin response, *M* value, AIR_arg_, and DI vs. PBO (*P*<0.05)
T2DM, drug naïve [157]	ExBID or PBO on MET background for 12 mo	Tests: HOMA-B, HOMA-IR, proinsulin-to-insulin ratio, combined euglycemic-hyperinsulinemic and hyperglycemic clamp with arginine stimulation, first- and second-phase insulin secretion, *M* value, DI ExBID improved HOMA-B and HOMA-IR vs. PBO (*P*<0.05). No change in proinsulin-to-insulin ratio vs. PBO Clamp: ExBID improved *M* value (+34%) and DI (+55%) vs. PBO (*P*<0.05) Clamp: ExBID improved first- (+21%) and second-phase (+34%) insulin response, and AIR_arg_ (+25%) vs. PBO (*P*<0.05)
T2DM [151]	ExBID or ROSI on MET background for 20 wk	MTT PPG, PP insulin, and PP C-peptide decreased in all groups vs. BL (*P*<0.05) MTT: ExBID and ExBID+ROSI improved Matsuda index and DI vs. BL (*P*<0.05). For ROSI, Matsuda index improved vs. BL (*P*<0.05), but no change in DI vs. BL MTT: ExBID had a greater first-phase insulin response than ROSI (*P*=0.018) MTT: ExBID and ExBID+ROSI: Greater second-phase insulin response than ROSI (*P*<0.05) Clamp: ROSI and ExBID+ROSI: Improved *M* value vs. BL (*P*<0.05). No change with ExBID

Abbreviations: ALO, alogliptin; BCF, β-cell function; BID, twice daily; BL, baseline; BMI, body mass index; d, day(s); DI, disposition index; Esc, dose escalation allowed; Ex, exenatide; FPG, fasting plasma glucose; HbA_1c_, glycated hemoglobin A_1c_; HOMA, homeostasis model assessment; IGI, insulinogenic index; IGT, impaired glucose tolerance; IR, insulin resistance; ISI, insulin sensitivity index; ISR, insulin secretion rate; IVGTT, intravenous glucose tolerance test; LINA, linagliptin; mo, month(s); MET, metformin; MTT, mixed-meal tolerance test; OGTT, oral glucose tolerance test; PBO, placebo; PIO, pioglitazone; PP, postprandial; PPG, postprandial glucose; QD, once daily; QW, once weekly; ROSI, rosiglitazone; SAXA, saxagliptin; SITA, sitagliptin; T2DM, type 2 diabetes mellitus; VILDA, vildagliptin; vs., versus; wk, week(s); y, year(s).

## ABBREVIATIONS

ADA: = American Diabetes Association
ALO: = Alogliptin
ARG stim: = Arginine stimulation
AUC: = Area under the curve
BCF: = β-cell function
BID: = Twice daily
BMI: = Body mass index
CGI: = Combined glucose intolerance
CVD: = Cardiovascular disease
d: = Day(s)
D/E: = Diet and exercise
DI: = Disposition index
DPPI: = Dipeptidyl peptidase-4 inhibitor
EGP: = Endogenous glucose production
Esc: = Dose escalation allowed
Ex: = Exenatide
FPG: = Fasting plasma glucose
GIP: = Glucose-dependent insulinotropic polypeptide
GLARG: = Insulin glargine
GLIB: = Glibenclamide
GLIC: = Gliclazide
GLIM: = Glimepiride
GLP-1: = Glucagon-like peptide-1
GLP-1RA: = Glucagon-like peptide-1 receptor agonist
GLY: = Glyburide
h: = Hour(s)
HbA_1c_: = Glycated hemoglobin A_1c_
HOMA: = Homeostasis model assessment
IFG: = Impaired fasting glucose
IGI: = Insulinogenic index
IGT: = Impaired glucose tolerance
IR: = Insulin resistance
ISI: = Insulin sensitivity index
ISR: = Insulin secretion rate
IVGTT: = Intravenous glucose tolerance test
LINA: = Linagliptin
LIRA: = Liraglutide
LIXI: = Lixisenatide
MET: = Metformin
MI: = Matsuda index
min: = Minute(s)
MTD: = Maximum tolerated dose
MTT: = Meal tolerance test
NA: = Not applicable
NG: = Not given
NGT: = Normal glucose tolerance
NS: = Not statistically significant; *P*>0.05
OAD: = Oral antidiabetes drug(s)
OGIS: = Oral glucose insulin sensitivity
OGTT: = Oral glucose tolerance test
PBO: = Placebo
PIO: = Pioglitazone
PP: = Postprandial
PPG: = Postprandial glucose
QD: = Once daily
QW: = Once weekly
ROSI: = Rosiglitazone
RPG: = Repaglinide
SAXA: = Saxagliptin
SFU: = Sulfonylurea(s)
SITA: = Sitagliptin
TID: = Three times a day
TROG: = Troglitazone
TZD: = Thiazolidinedione(s)
VILDA: = Vildagliptin
vs.: = Versus
wk: = Week(s)
y: = Year(s)
